# Isthmin-1 attenuates allergic Asthma by stimulating adiponectin expression and alveolar macrophage efferocytosis in mice

**DOI:** 10.1186/s12931-023-02569-1

**Published:** 2023-11-06

**Authors:** Jong Huat Tee, Udhaya Vijayakumar, Mahalakshmi Shanmugasundaram, Terence Y. W. Lam, Wupeng Liao, Yuansheng Yang, W. S. Fred Wong, Ruowen Ge

**Affiliations:** 1 Department of Biological Sciences, Faculty of Science, National University of Singapore, Singapore, 117543 Singapore; 2 Department of Pharmacology, Yong Loo Lin School of Medicine, National University of Singapore, Singapore, 117600 Singapore; 3 Bioprocessing Technology Institute, A*STAR, Singapore, 138668 Singapore; 4 Singapore-HUJ Alliance for Research and Enterprise (SHARE), National University of Singapore, Singapore, 138602 Singapore; 5 Drug Discovery and Optimization Platform, Yong Loo Lin School of Medicine, National University Health System, Singapore, 117600 Singapore

**Keywords:** Isthmin-1, Asthma, Adiponectin, Efferocytosis, Necroptosis, Alveolar macrophage, Type 2 alveolar epithelial cells, Airway remodeling, RNA sequencing, GRP78

## Abstract

**Background:**

Allergic asthma is a common respiratory disease that significantly impacts human health. Through in silico analysis of human lung RNASeq, we found that asthmatic lungs display lower levels of Isthmin-1 (ISM1) expression than healthy lungs. ISM1 is an endogenous anti-inflammatory protein that is highly expressed in mouse lungs and bronchial epithelial cells, playing a crucial role in maintaining lung homeostasis. However, how ISM1 influences asthma remains unclear. This study aims to investigate the potential involvement of ISM1 in allergic airway inflammation and uncover the underlying mechanisms.

**Methods:**

We investigated the pivotal role of ISM1 in airway inflammation using an ISM1 knockout mouse line (*ISM1*^−/−^) and challenged them with house dust mite (HDM) extract to induce allergic-like airway/lung inflammation. To examine the impact of ISM1 deficiency, we analyzed the infiltration of immune cells into the lungs and cytokine levels in bronchoalveolar lavage fluid (BALF) using flow cytometry and multiplex ELISA, respectively. Furthermore, we examined the therapeutic potential of ISM1 by administering recombinant ISM1 (rISM1) via the intratracheal route to rescue the effects of ISM1 reduction in HDM-challenged mice. RNA-Seq, western blot, and fluorescence microscopy techniques were subsequently used to elucidate the underlying mechanisms.

**Results:**

ISM1^−/−^ mice showed a pronounced worsening of allergic airway inflammation and hyperresponsiveness upon HDM challenge. The heightened inflammation in *ISM1*^−/−^ mice correlated with enhanced lung cell necroptosis, as indicated by higher pMLKL expression. Intratracheal delivery of rISM1 significantly reduced the number of eosinophils in BALF and goblet cell hyperplasia. Mechanistically, ISM1 stimulates adiponectin secretion by type 2 alveolar epithelial cells partially through the GRP78 receptor and enhances adiponectin-facilitated apoptotic cell clearance via alveolar macrophage efferocytosis. Reduced adiponectin expression under ISM1 deficiency also contributed to intensified necroptosis, prolonged inflammation, and heightened severity of airway hyperresponsiveness.

**Conclusions:**

This study revealed for the first time that ISM1 functions to restrain airway hyperresponsiveness to HDM-triggered allergic-like airway/lung inflammation in mice, consistent with its persistent downregulation in human asthma. Direct administration of rISM1 into the airway alleviates airway inflammation and promotes immune cell clearance, likely by stimulating airway adiponectin production. These findings suggest that ISM1 has therapeutic potential for allergic asthma.

**Graphical abstract:**

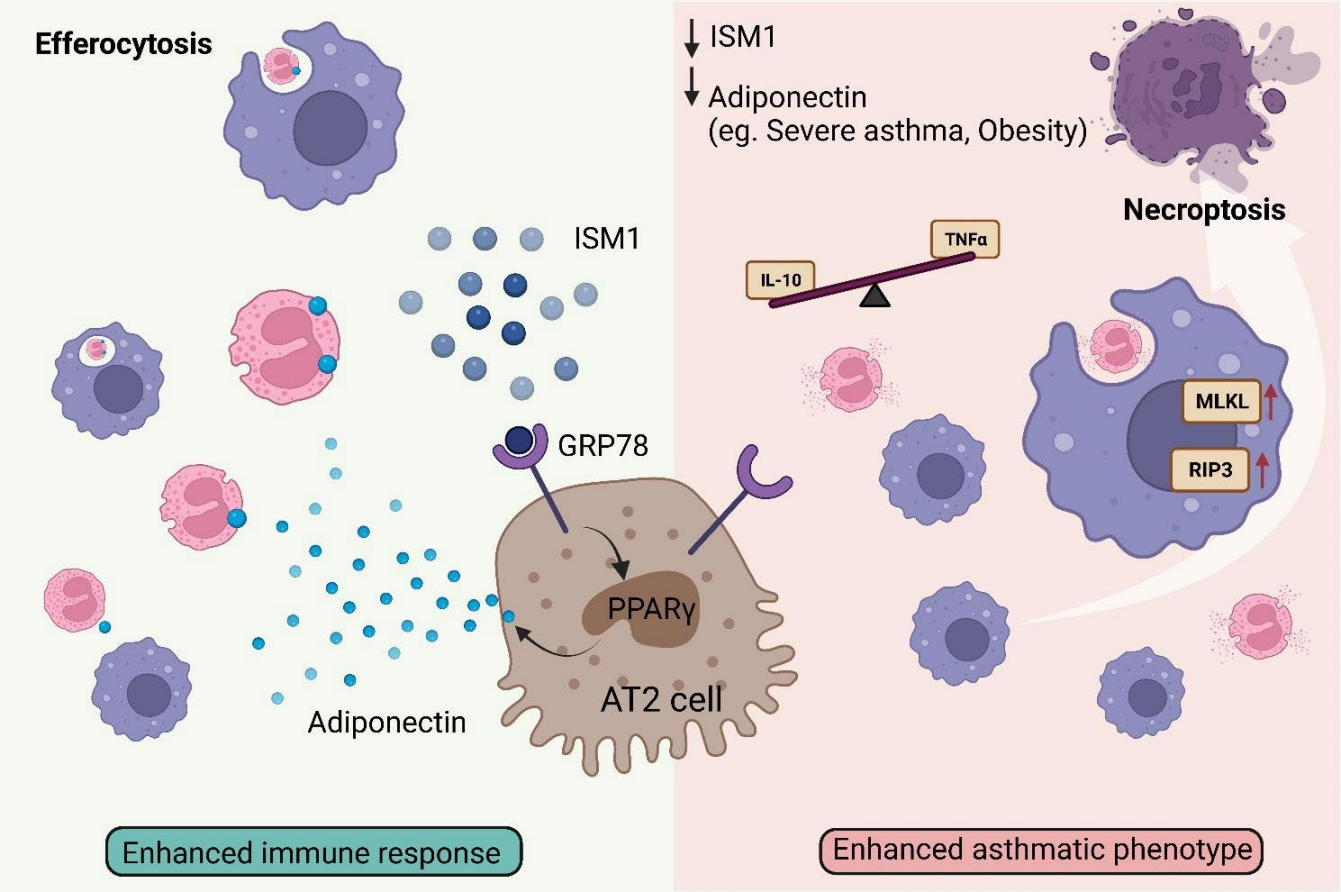

**Supplementary Information:**

The online version contains supplementary material available at 10.1186/s12931-023-02569-1.

## Background

Asthma is a chronic airway inflammatory disorder associated with an aberrant immune response to allergens and tissue remodeling. Asthma symptoms include shortness of breath, coughing, and wheezing, which can vary in frequency and intensity over time [1]. This condition affects more than 300 million people worldwide and poses a major challenge to healthcare costs [2]. Asthma is thought to be caused by an abnormal inflammatory response to environmental agents in genetically susceptible individuals [3, 4]. Although advancements in asthma management have been made in recent years, severe asthma that does not respond to corticosteroids remains a significant challenge, and asthma-related mortality still occurs. Hence, there is a need for a more comprehensive understanding of the underlying pathobiology of asthma to develop new and effective therapeutic options.

During allergen-induced airway inflammation, the initial inflammatory response involves the activation of T helper type 2 (T_H_2) cells, which stimulate the influx of eosinophils, mast cells, and other leukocytes into the airways, along with increased serum IgE production. Subsequently, airway structural cells and resident immune cells release anti-inflammatory or pro-resolving mediators to control the extent of the immune response and resolve the inflammation and restore the airway back to its steady state. However, in asthmatic patients, the inflammatory process can become disrupted due to an overzealous inflammatory phase overproducing pro-inflammatory factors or the disruption of the anti-inflammatory phase. These can result in a loss of immune tolerance against common airborne allergens [5, 6].

Isthmin-1 (ISM1) is a protein highly expressed in the trachea and lungs of both mice and humans. Its expression in the airway epithelium was significantly enhanced by airway instillation of lipopolysaccharide (LPS), a bacterial endotoxin [7, 8]. ISM1 is also expressed by lung lymphocytes such as natural killer (NK) cells, NKT cells, and CD4^+^ T cells [9], suggesting a potential role in immune regulation. We previously identified ISM1 as a pro-apoptotic protein that functions through two cell surface receptors, αvβ5 integrin and cell-surface GRP78 (csGRP78) in endothelial cells [10, 11] and induces lung vascular permeability via the csGRP78 receptor and Src activation [12]. Recently, ISM1 has been shown to selectively induce apoptosis of pro-inflammatory alveolar macrophages (AMs) via the csGRP78 receptor, thereby maintaining lung homeostasis [13].

House dust mite (HDM) extract is known to induce endoplasmic reticulum (ER) stress in both human and mouse bronchial epithelial cells, which triggers the upregulation of the ER chaperone GRP78 [14]. This upregulation of GRP78 expression leads to increased csGRP78 in stressed cells [15, 16]. In allergic asthma, the bronchial epithelium is often stressed and damaged [17]. This airway epithelial damage is believed to initiate and orchestrate inflammatory responses by releasing chemokines and cytokines, which recruit and activate inflammatory immune cells. However, whether ISM1 plays a role in the pathogenesis of asthma remains unknown.

Herein, we investigated the role of ISM1 in allergic airway inflammation induced by HDM extract in mice. Our findings indicate that ISM1 deficiency exacerbates airway inflammation and airway hyperresponsiveness (AHR), whereas intratracheal (i.t.) delivery of recombinant ISM1 (rISM1) attenuates HDM-induced airway inflammation. RNASeq analyses of ISM1-deficient lungs revealed significant downregulation of lung adiponectin, an anti-inflammatory adipokine [18, 19]. Furthermore, we discovered that ISM1 potently stimulates adiponectin secretion in cultured human type 2 alveolar epithelial cells (A549) and enhances adiponectin-facilitated apoptotic cell efferocytosis by AMs both in vitro and in vivo [20]. Our work unveils a previously unknown link between ISM1, adiponectin, and allergic airway inflammation, providing novel molecular insights into the pathophysiology of allergic airway inflammation.

## Methods

### Mice

Wild-type (WT) C57BL/6J mice were purchased from InVivos Pte. Ltd., Singapore. ISM1 knockout (*ISM1*^*−/−*^) mice were generated in-house on a C57BL/6J background as described previously [8, 13]. Seven- to eight-week-old age-matched mice were used in this study. All mice were housed under pathogen-free conditions, with water and food pellets supplied *ad libitum*.

### HDM-induced allergic-like airway/lung inflammation

A well-established animal model of HDM-induced allergic-like airway/lung inflammation was adopted for this study [21]. Mice were sensitized on days 1, 7, and 14 with 50 µg of HDM extract (*Dermatophagoides pteronyssinus*) (Greer Laboratories) in 20 µL of normal saline via the intratracheal (*i.t.*) route after short anesthesia with isoflurane. Control mice received an equal volume of saline. For rISM1 treatment, 25 µL (1 mg/mL) of recombinant mouse ISM1 dissolved in 20 mM Tris buffered saline (TBS, pH 7.6) was administered intratracheally on day 12, 13, and 1 h before HDM challenge on day 14, 15, and 16. The vehicle group received an equal volume of 20 mM TBS. All mice were euthanized on day 17, and blood was collected by cardiac puncture to measure total IgE and HDM-specific IgE in the serum. Bronchoalveolar lavage fluid (BALF) was collected by perfusing 0.5 mL of chilled PBS into the trachea through a 22-inch IV catheter, which was repeated three times to gather approximately 1.5 mL of BALF. The right lung was excised for protein and RNA extraction, whereas the left lung was fixed in 10% formalin and paraffin-embedded for histological sectioning. Serum, BALF, and the right lung were snap-frozen in liquid nitrogen and stored at -80 °C until use.

### Assessment of airway hyperresponsiveness

An independent set of animals was allocated for the invasive airway hyperresponsiveness (AHR) measurement using a Buxco® FinePointe resistance and compliance system equipped with analysis software (Data Sciences International, Harvard Bioscience). AHR was induced with methacholine 3 days after the final HDM sensitization. Briefly, the mice were anesthetized with a combination of ketamine (75 mg/kg) and medetomidine (1 mg/kg). A tracheotomy was performed and the cannulated mice were transferred into a whole-body chamber and mechanically ventilated. Animals were acclimatized for 5 min before being aerosolized with PBS. The baseline lung resistance against the PBS challenge was recorded for 3 min. Then, an increasing concentration of methacholine (5, 10, 20, and 40 mg/ml) was aerosolized and lung resistance was recorded. The average lung resistance (R_L_) and dynamic compliance (C_dyn_) values were used to demonstrate the changes in the lung function of the mice.

### Enzyme-linked immunosorbent assay (ELISA)

Cytokines were quantified using LUNARIS™ mouse 12-plex cytokine kit (LMCY-20,120 S, Ayoxxa Biosystems) and MILLIPLEX® Mouse Th17 Magnetic Bead Panel (MTH17MAG-47 K, Millipore) according to the manufacturer’s protocol. Chemokines such as eotaxin-1, eotaxin-2, and monocyte chemoattractant protein-1 were measured by singleplex ELISA (Raybiotech). The serum levels of total IgE and HDM-specific IgE were measured using an OptEIA™ Mouse IgE ELISA kit (555,248, BD Biosciences) according to the manufacturer’s protocol. ISM1 was measured in mouse BALF using a LEGEND MAX™ Mouse Isthmin ELISA Kit (438,907, BioLegend). TGFβ_1_ in BALF and lung tissue lysates was measured using a TGFβ_1_ ELISA kit (DY1679, R&D Systems). Adiponectin levels were measured using a mouse adiponectin/Acrp30 Quantikine ELISA kit (R&D Systems).

### Histopathologic evaluation of lung tissue

The left lung tissue section was stained with hematoxylin and eosin (H&E) and periodic acid-Schiff (PAS) to evaluate airway inflammation and goblet cell metaplasia, respectively. Arbitrary scoring was performed by three experienced observers who were blinded to the treatment group. The extent of peribronchial and perivascular cell infiltration and mucus-producing goblet cells in the lung tissue were quantified on a 0–4 scale as described previously [22]. Inflammatory score: 0 = no infiltration; 1 = a few cells; 2 = a ring of cells with one-layer of cell depth; 3 = a ring of cells with two to four-layer of cell depth; 4 = a ring of cells with more than four layers of cell depth. The number of PAS-positive cells was counted under a light microscope, and the results were expressed as the percentage of PAS-positive cells over the total number of epithelial cells in the airway. PAS score: 0 = no PAS-positive cell; 1 = less than 25% positive cells; 2 = 50% positive cells; 3 = 75% positive cells; and 4 = more than 75% positive cells. The inflammatory and goblet cells were scored in at least six different fields for each lung section. Mean scores were obtained from five animals.

### Immunohistochemistry and immunofluorescence staining

Lung Sections (5 μm) were deparaffinized and rehydrated and antigen was retrieved by heating in sodium citrate buffer (10 mM sodium citrate, 0.05% Tween 20, pH 6.0). After quenching the endogenous peroxidase activity with 3% hydrogen peroxide, the sections were blocked with 3% BSA, stained with the respective primary and secondary antibodies and reacted with diaminobenzidine substrate. Nuclei were counterstained with hematoxylin. Immunofluorescence-stained slides were counterstained with DAPI. Images were captured with a Zeiss Axio Imager M2 microscope and analyzed using ImageJ (NIH).

### Flow cytometry

Total and differential cell counts in the BALF were determined by Cytoflex LX flow cytometer with CytExpert software ver.2.3 (Backman Coulter). Forward and side scatter plots were used to exclude cell debris and clumps. Total leukocytes were identified as CD45^+^, AMs as CD11c^+^ Siglec-F^+^, eosinophils as CD11c^−^ Siglec-F^+^, neutrophils as GR-1^+^ CD11b^+^, and dendritic cells as CD11c^+^ CD11b^+^. All antibodies used for flow cytometry were purchased from Miltenyi Biotec. A sequential gating strategy was performed to differentiate among different cell types.

### Western blotting

Frozen lung tissues were ground in chilled 8 molar urea lysis buffer containing protease and phosphatase inhibitor cocktail (Roche). On the other hand, protein in the cell culture conditioned media was precipitated using cold acetone (-20 °C). Palleted protein was then resolubilized in electrophoresis sample buffer. Protein lysates (30 µg protein) were electrophoretically separated on either 10% SDS PAGE glycine gels or 15% tricine gels and transferred to a nitrocellulose membrane. After blocking with 5% BSA, the membranes were sequentially incubated with the primary antibody and secondary antibody. Finally, the protein bands were analyzed using the LI-COR Odyssey Imaging system. The primary antibodies used were as follows: rabbit anti-cleaved TGFβ_1_ (V) (sc-146, SCBT), mouse anti-RIP3 (B-2) (sc-374,639, SCBT), rabbit anti-pMLKL (D6E3G, CST), rabbit anti-caspase 8 (D35G2, CST), rabbit anti-cleaved caspase 3 (D175, CST), rabbit anti-PPARγ (16,643-AP, Proteintech), rabbit anti-adiponectin (21,613-AP, Proteintech), goat anti-adiponectin (AF1119, R&D Systems), and mouse anti-β-actin (C4) (sc47778, SCBT) was used as the loading reference. The secondary antibodies used were as follows: donkey anti-mouse IRDye 680, donkey anti-rabbit IRDye 800, and donkey anti-goat IRDye 800 (all from LI-COR Biosciences). For the antibody neutralization experiment, the following antibodies were used: mouse anti-GRP78 (A10) (sc-376,768, SCBT), mouse anti-αvβ5 (P1F76, SCBT).

### RNA sequencing

Total RNA was isolated from perfused whole lungs using TRIzol reagent (Invitrogen) according to the manufacturer’s recommended protocol. Strand-specific mRNA-seq libraries for the Illumina NovaSeq 6000 platform were generated and sequenced by Novogene, Singapore. Briefly, high-quality total RNA with an RNA integrity number (RIN) > 8 was subjected to poly-A enrichment and a directional mRNA library was prepared. The library was then subjected to paired-end sequencing of 150 bp read length and sequence depth of 40 million reads per sample. The raw sequencing data were filtered to remove low-quality reads and adaptor sequences. Clean reads were mapped onto the reference genome *Mus musculus* (GRCm38/mm10) using STAR software. The number of reads that mapped a certain gene or transcript was measured to calculate the gene expression level and the raw counts obtained were normalized to FPKM (fragments per kilobase of transcript sequence per million base pairs sequenced), which took sequence depth and gene length into account. Pearson correlation and principal component analysis (PCA) were performed using normalized read counts.

For initial differential expression analysis, the read counts from the gene expression level analysis were input into DESeq2 with the following thresholds: P_adj_ ≤ 0.05, log_2_FC ≥ 0. For subsequent analysis, the biomaRt R package was used to convert the ensemble ID to a mouse gene symbol and to obtain the gene types. Protein-coding genes were chosen for further analysis. Genes with mean counts per million (CPM) across samples less than 2 were filtered out using the CPM function from the edgeR package. Only protein-coding genes with CPM > 2 were input into DESeq2 for differential expression analysis with the following parameters: log_2_FC ≤ -1 or log_2_FC ≥ 1 & P_adj_ < 0.05 to remove low expressing genes and to identify highly differentiated genes. Deseq2-normalized expression values were plotted as heatmaps and volcano plots using the heatmap R package and volcano R package respectively. For pathway enrichment analysis, the Fgsea R package was used with the mouse gene ortholog version of the Kyoto Encyclopedia of Genes (KEGG) and REACTOME gene sets from MsigDB database V5.2. Pathways with a Benjamini-Hochberg P_adj_ < 0.05 were considered significantly deregulated pathways for each gene set. Gene Ontology (GO) and KEGG pathway analyses of DEGs were performed using DAVID software. The *p*-value was calculated by the Benjamini-Hochberg correction procedure for multiple hypothesis testing.

### qRT-PCR

Total RNA (1 µg) from lung tissues was reverse transcribed using a Maxima First Strand cDNA Synthesis kit (Thermo Scientific) according to the manufacturer’s instructions. For qPCR, SsoAdvanced Universal SYBR Green Supermix (Bio-Rad) was used. The primer sequences used are listed in Additional file 1: Table S1. The relative quantification of gene expression was determined using the comparative Cq (ΔΔCq) method and represented as the Log_2_ fold change.

### Efferocytosis Assay

In vitro efferocytosis assay was performed according to a previously described protocol with minor modifications [23]. Jurkat T cells were exposed to UV irradiation (254 nm) for 30 min and incubated in complete RPMI media supplemented with 10% FBS for 2 h to induce early apoptosis. Apoptotic Jurkat T cells were then labeled with Phrodo™ Red, succinimidyl ester (P36600, Thermo Scientific) for 1 h according to the manufacturer’s protocol. Labeled-apoptotic Jurkat T cells (1 × 10^6^ cells) were treated with recombinant adiponectin (HY-P7129, MedChemExpress) or A549 conditioned media treated with (CM^+ ISM1^) or without rISM1 (CM^− ISM1^) for 1 h and then added to MH-S cells at a 1:10 ratio (Jurkat: MH-S) in Opti-MEM I reduced serum medium. The serum-free condition was used to nullify the contribution of adiponectin in serum. Efferocytosis was detected and measured using the IncuCyte ZOOM live cell imaging system (Sartorius) [24]. ISM1-induced adiponectin in A549 conditioned media was then neutralized using an anti-human adiponectin/Acrp30 polyclonal antibody (AF1065, RnD Systems) prior to the efferocytosis assay. In some experiments, apoptotic Jurkat T cells were labeled with CellTracker™ Green CMFDA (5-chloromethyl fluorescein diacetate) (Invitrogen). Quantifications were carried out using six microscopic fields per well, and efferocytosis capacity was expressed as a percentage of cells containing fluorescent-labeled apoptotic bodies over the total number of MH-S cells in the field.

In vivo efferocytosis assay was performed as described earlier [25]. Briefly, Phrodo™ Red-labeled early apoptotic Jurkat T cells were instilled into WT and *ISM1*^*−/−*^ mouse lungs via intratracheal delivery. BALF was collected 90 min after the mouse had woken up from anesthesia while dosing with the apoptotic cells. The macrophage population in the BALF was stained with FITC-conjugated CD68 antibody (Clone FA-11, Biolegend). Efferocytosis capacity was assessed by flow cytometry, where CD68-Phrodo double-positive cells were identified as phagocytotic macrophages.

### Statistical analysis

Results are presented as the mean ± SD. Data were analyzed with Student’s t test or one-way ANOVA, followed by a post hoc multiple comparison test with GraphPad Prism version 9 software. Error bars represent the standard deviation of the mean. A *P*-value < 0.05 was considered statistically significant. The levels of statistical significance were denoted at *P < 0.05, **P < 0.01, ***P < 0.001, ****P < 0.0001, and *n.s.* non-significant.

## Results

### ISM1 is downregulated in Asthma

**Fig. 1:**
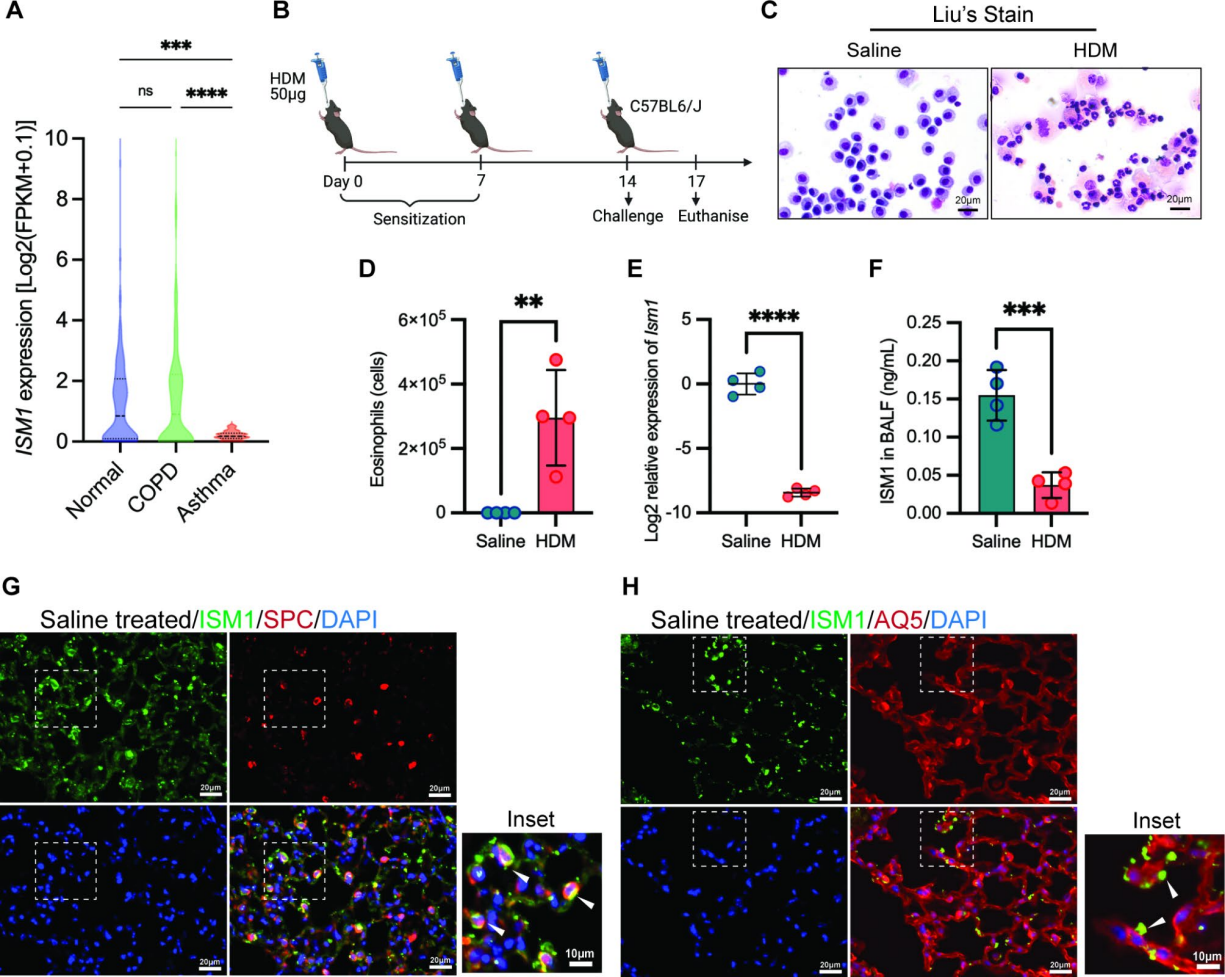
ISM1 expression is downregulated under asthmatic conditions. **(A)** *ISM1* expression in human lung tissue. RNA-Seq data were obtained and analyzed from the public databases Genotype-Tissue Expression B37 and B38_GC33 projects using Ingenuity Pathway Analysis software (OmicsSoft, Qiagen). **(B)** Timeline for allergen exposure in the HDM-induced allergic asthma mouse model (n = 4). **(C)** Representative photomicrographs of Liu’s staining of BALF cells showing eosinophil predominance in the HDM-sensitized mouse lungs. Bars = 20 µm. **(D)** Quantification of eosinophils in the HDM-sensitized mouse lungs by flow cytometry. **(E)** Validation of *Ism1* gene expression by qRT-PCR in HDM-challenged mouse lungs compared to the saline control group. **(F)** Secreted ISM1 levels in BALF measured by sandwich ELISA. **(G)** Immunofluorescence staining showing colocalization of ISM1 with surfactant protein C (SPC) marker in normal lung sections. **(H)** Expression of ISM1 appears in proximity to aquaporin 5 (AQ5) staining of normal lung sections. Bars = 20 µm and 10 µm (inset). Data are presented as the mean ± SD. **P < 0.01; ***P < 0.001; ****P < 0.0001; using Student’s t test. FPKM, fragments per kilobase of transcript per million mapped reads; HDM, house dust mite; BALF, bronchoalveolar lavage fluid; DAPI, 4’,6-diamidino-2-phenylindole

To explore the potential role of ISM1 in asthma, we conducted an analysis of human RNA-Seq data from the Genotype-Tissue Expression B37 and B38_GC33 projects using Ingenuity Pathway Analysis (IPA) (OmicsSoft, Qiagen). While *ISM1* expression has a bimodal distribution in the human population (Fig. 1A and Additional file 1: Table S2), we observed consistent downregulation of ISM1 expression in asthma with a unimodal distribution (Additional file 2: Fig. S1). To determine whether *Ism1* expression follows a similar trend in mouse allergic-like airway/lung inflammation, we induced acute eosinophilic asthma with a clinically relevant house dust mite (HDM, *Dermatophagoides pteronyssinus*) allergen (Fig. 1B) [26]. HDM-challenged mice showed massive eosinophil infiltration into the airway, a pathological hallmark of the main type of human asthma (Fig. 1, C, D). Our results indicate that *Ism1* mRNA expression is significantly downregulated in HDM-challenged C57BL/6J mouse lungs, while ISM1 protein is significantly reduced in BALF (Fig. 1, E, F).

Previous studies have shown that ISM1 is expressed in mouse bronchial epithelium and AMs [7, 12, 13]. Here, we demonstrated that a considerable proportion of intracellular ISM1 colocalized with surfactant protein C (SPC), a marker for alveolar type 2 epithelial cells (AT2) and was in proximity to aquaporin 5 (AQ5), a marker for alveolar type 1 epithelial cells, in lung tissue (Fig. 1, G, H). These findings indicate that ISM1 expression is downregulated in asthma and that AT2 cells may be one of the cell types expressing ISM1.

### Allergic airway inflammation is heightened in *ISM1*^*−/−*^ mice

**Fig. 2:**
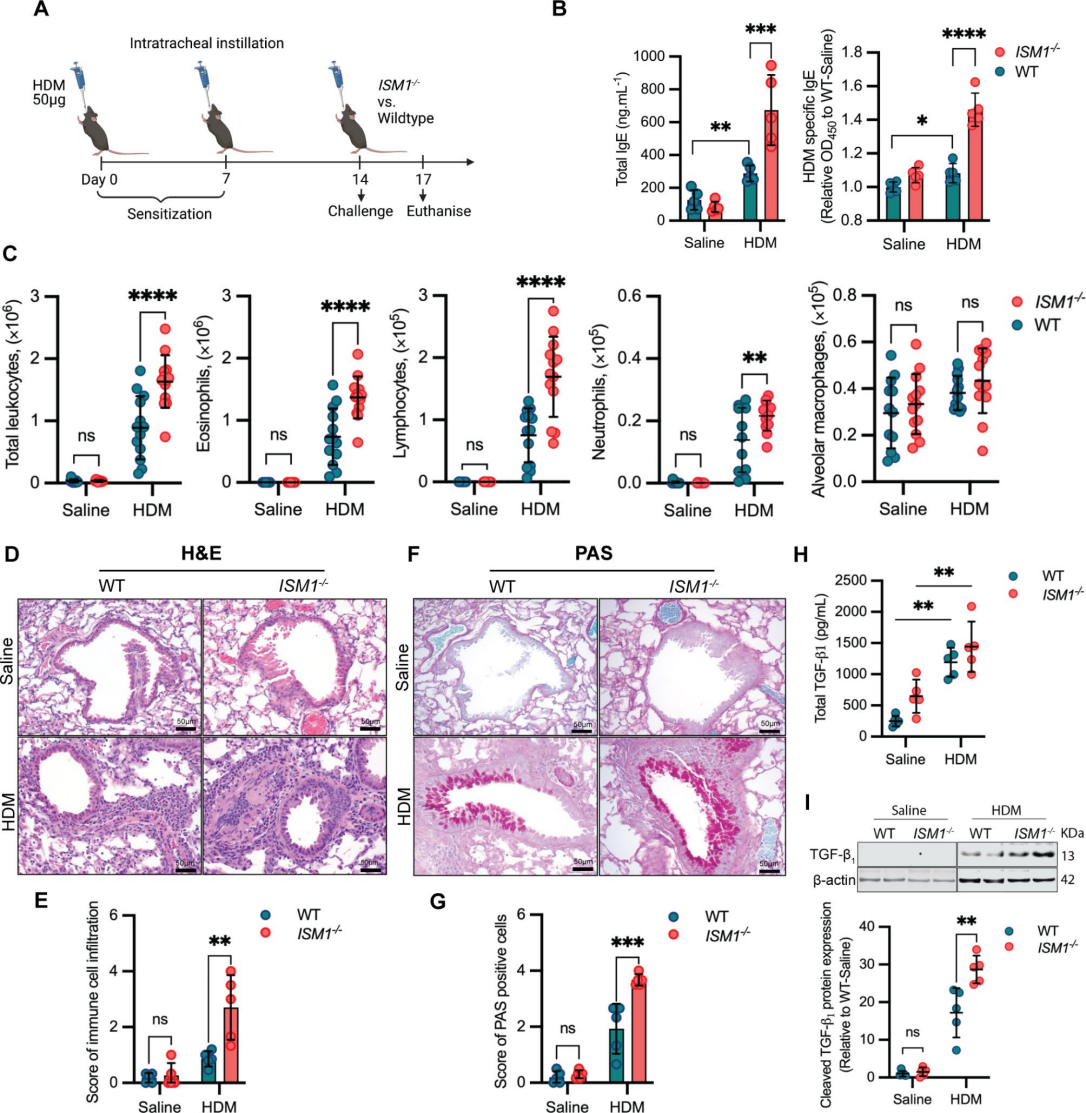
ISM1 deficiency exacerbates HDM-induced allergic asthma phenotypes. **(A)** Sensitization protocol of HDM-induced asthma in WT and *ISM1*^*−/−*^ mice. Animals were sensitized on days 0, 7, and 14 with 50 µg of HDM extract and euthanized on day 17. **(B)** Serum titer for total and HDM-specific IgE comparing WT (n = 6) and *ISM1*^*−/−*^ (n = 8) mice with or without HDM sensitization. **(C)** Cellular composition of BALF measured by flow cytometry (n = 13). **(D-E)** Representative photomicrographs of H&E staining of lung tissue sections along with the immune cell infiltration score between WT (n = 5) and *ISM1*^*−/−*^ (n = 5) mice with or without HDM sensitization. Bars = 50 μm. **(F-G)** Representative photomicrographs of PAS staining of the lung tissue section along with the PAS-positive cell score between WT (n = 5) and *ISM1*^*−/−*^ (n = 5) mice with or without HDM sensitization. Bars = 50 μm. **(H)** Total TGFβ_1_ levels in the BALF quantified using ELISA (n = 5). **(I)** Western blot analysis of cleaved TGFβ_1_ expression in lung tissues (n = 5). Data are presented as the mean ± SD. **P < 0.01; ***P < 0.001; ****P < 0.0001, using one-way ANOVA. H&E, hematoxylin and eosin; PAS, Periodic acid-Schiff, TGFβ_1_, transforming growth factor beta 1

To investigate the role of ISM1 in allergic asthma, we conducted a study using HDM-induced allergic-like airway/lung inflammation in *ISM1*^*−/−*^ and wild-type (WT) mice. The sensitization of mice was performed by intratracheal administration of HDM extract on day 1, 7, and 14, as described previously (Fig. 2A) [26]. As a control, mice were administered an equal volume of saline. All mice were euthanized three days after the last sensitization, and samples (BALF, serum, and lung tissue) were collected for analysis. As shown in Fig. 2B, both total IgE and HDM-specific IgE levels in the sera were significantly higher in *ISM1*^*−/−*^ mice under HDM-induced allergic asthma than those of the WT mice.

Consistent with previous reports, after HDM challenge, eosinophils became the predominant cell type in the BALF of both *ISM1*^*−/−*^ and WT mice, indicating eosinophil-mediated airway inflammation (Fig. 2C). AMs were the predominant cells in the control mice of both groups (Additional file 3: Fig. S2, A, B). Notably, FACS analysis revealed a close to 2-fold increase in total infiltrating leukocytes, eosinophils, lymphocytes, and neutrophils in the BALF of *ISM1*^*−/−*^ mice compared with that of the WT mice under HDM challenge (Fig. 2C). This observation was further confirmed by Liu’s staining of BAL cells (Additional file 3: Fig. S2C). Additionally, HDM challenge activated AMs, as evidenced by their enlarged size (Additional file 3: Fig. 2C). Histological analyses of lung sections showed extensive infiltration of eosinophils and mononuclear inflammatory cells in the peribronchial and perivascular regions in both WT and *ISM1*^*−/−*^ mice (Fig. 2, D, E), concomitant with mucus hypersecretion, as shown by PAS staining (Fig. 2, F, G). These phenotypes were more pronounced in *ISM1*^*−/−*^ mouse lungs under HDM challenge. Notably, we observed spontaneous emphysema phenotype in *ISM1*^*−/−*^ mouse lungs consistent with our earlier findings (Additional file 3: Fig. S2D) [8, 13], suggesting that the pre-existence of lung inflammation in the absence of ISM1 could potentiate the airway response against the allergen. Consistent with our previous observations, the chronic lung inflammation phenotype under *Ism1* deficiency was milder in the C57BL/6J background, with the increase in AM number only obvious in a fraction of the mice (Fig. 2C). Nevertheless, the emphysema phenotype is consistent with that in *Ism1*^*−/−*^ FVB/N mice and has been attributed to more AMs being in the activated proinflammatory state [8, 13].

Transforming growth factor-beta 1 (TGFβ_1_), a major mediator of airway remodeling is linked with asthma severity [27]. Our results showed that the total TGFβ_1_ level in the BALF was significantly increased in both WT and *ISM1*^*−/−*^ mice upon HDM sensitization (Fig. 2H). Because TGFβ peptides are abundantly synthesized as inactive latent precursors, TGFβ activation is recognized as a critical step in the control of their bioactivity [28]. Hence, we investigated the expression of cleaved TGFβ_1_ in the lung lysates. Interestingly, we found that cleaved active TGFβ_1_ expression in the lung lysates was further elevated 1.6-fold in *ISM1*^*−/−*^ mice compared with WT mice (Fig. 2H). These data suggest that ISM1 plays a crucial role in inflammation suppression and immune regulation in the airway and lungs. ISM1 deficiency exacerbates the immune response and enhances airway remodeling in HDM-induced allergic airway inflammation.

### *ISM1*^*−/−*^ mice present altered cytokine/chemokine responses and airway hyperresponsiveness to HDM

**Fig. 3:**
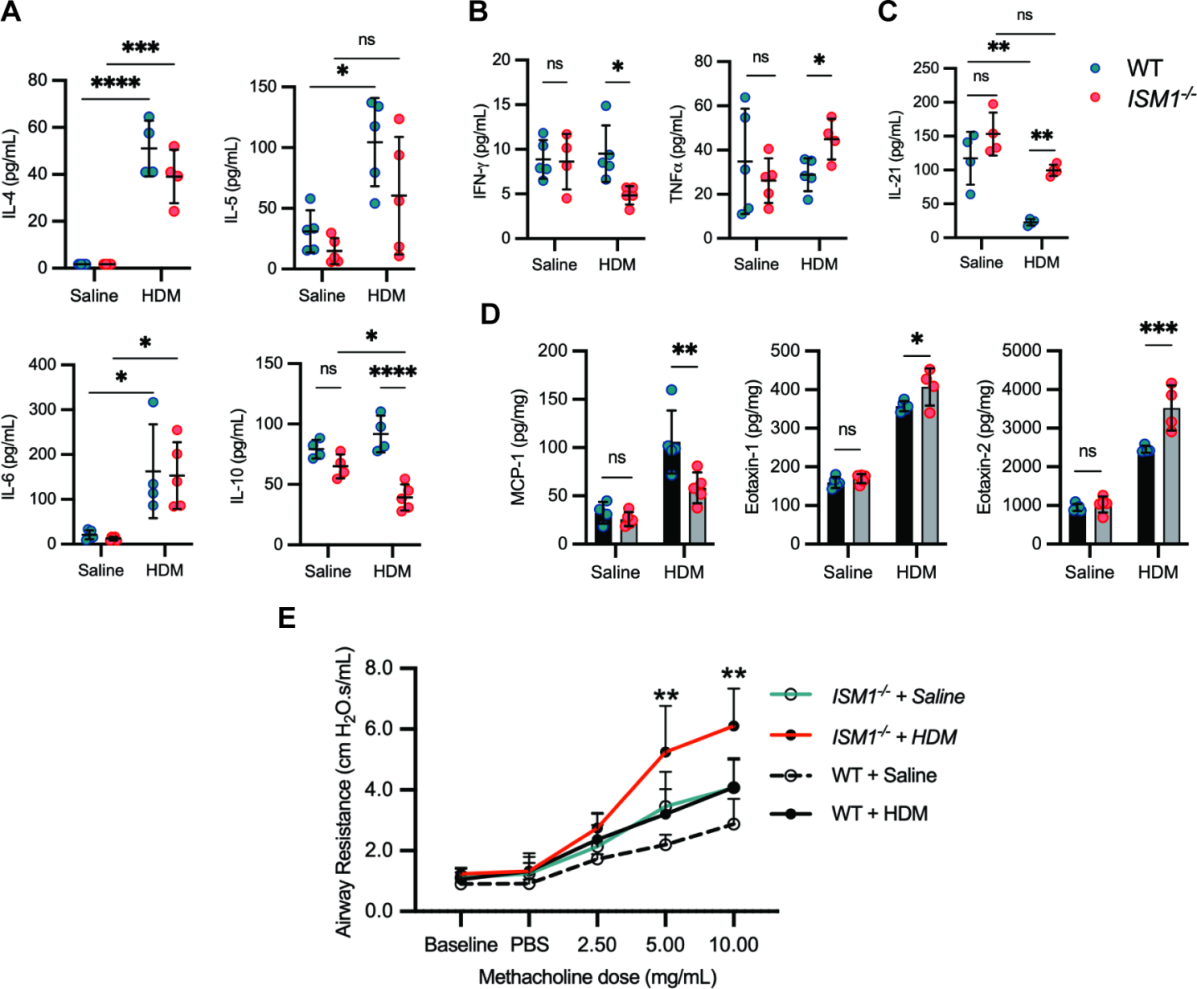
ISM1 deficiency exacerbates HDM-induced airway hyperresponsiveness. **(A)** Levels of T_H_2 cytokines in the BALF (n = 4–5). **(B)** Levels of T_H_1 cytokines in the BALF (n = 4–5). **(C)** Levels of T_H_17 cytokines in the BALF (n = 4–5). **(D)** Levels of chemokines in the lung tissue lysates (n = 4–5). Data are presented as the mean ± SD. *P < 0.05; **P < 0.01; ***P < 0.001; ****P < 0.0001, using one-way ANOVA. **(E)** Assessment of AHR in WT and *ISM1*^*−/−*^ mice with or without HDM sensitization. Airway resistance was measured in response to increasing concentrations of methacholine (0, 2.5, 5, and 10 mg/mL) in PBS (n = 6). Data are presented as the mean ± SD. **P < 0.01, using two-way ANOVA. IL, interleukin; IFNγ, interferon-gamma; TNFα, tumor necrotic factor alpha; MCP-1, monocyte chemoattractant protein 1, PBS, phosphate-buffered saline

To further investigate the impact of ISM1 deficiency on immune regulation, we measured cytokine and chemokine levels in BALF and lung lysates. Among the T_H_2 cytokines, IL-10 was significantly lower in *ISM1*^*−/−*^ mice upon HDM challenge, about 2-fold lower than that in HDM-challenged WT mice (Fig. 3A). Both IL-4 and IL-5, which are crucial in eosinophil recruitment and maturation, were similarly increased in the BALF of HDM-sensitized WT and *ISM1*^*−/−*^ mice compared to the saline control (Fig. 3A). IL-6 levels were also similarly stimulated, consistent with the infiltration of inflammatory cells such as macrophages and neutrophils upon HDM sensitization. However, the levels of IL-4, IL-5, and IL-6 did not differ between HDM-sensitized WT and *ISM1*^*−/−*^ mice. IL-13 levels were similar in both WT and *ISM1*^*−/−*^ mice, regardless of sensitization (Additional file 4: Fig. S3A).

In HDM-sensitized *ISM1*^*−/−*^ mice, IFNγ, a pro-resolution T_H_1 cytokine [29], was significantly decreased, while TNFα levels were slightly increased (Fig. 3B). Although statistically insignificant, the levels of other T_H_1 cytokines such as IL-1β, IL-2, and IL-12p70, were also lower in sensitized *ISM1*^*−/−*^ mice (Additional file 4: Fig. S3B). IL-21, which is highly produced by T_H_17 cells and has been reported to promote apoptosis of Treg cells and inhibit the development of IFNγ-producing T_H_1 cells in allergic airway inflammation [30–32], was significantly reduced upon HDM challenge in WT mice but remained relatively high in the BALF of HDM-sensitized *ISM1*^*−/−*^ mice (Fig. 3C). No difference was observed in the level of IL-17 A, a cytokine implicated in neutrophilic asthma [33] (Additional file 4: Fig. S3C).

The effect of ISM1 deficiency on chemokines was also investigated. Monocyte chemoattractant protein 1 (MCP-1/CCL2), which is involved in neutrophil and macrophage recruitment [34], was found to be reduced in the lung lysates of *ISM1*^*−/−*^ mice, indicating that ISM1 deficiency does not specifically enhance neutrophilic asthma (Fig. 3D). Conversely, eotaxin-1 and eotaxin-2, which are potent eosinophil chemoattractants [35], were increased in the lung lysates of sensitized *ISM1*^*−/−*^ mice (Fig. 3D). Notably, HDM challenge also significantly increased the BALF levels of eotaxin-1 and eotaxin-2 (Additional file 4: Fig. S3D), although no further differences were observed between *ISM1*^*−/−*^ and WT mice. These findings indicate that ISM1 deficiency alters the levels of multiple cytokines/chemokines, which could influence airway inflammation.

Accordingly, our results showed that ISM1 deficiency significantly enhanced airway hyperresponsiveness (AHR), a pathological feature of asthma. Airway resistance (R_L_) and dynamic compliance (C_dyn_) were evaluated in response to increasing concentrations of methacholine in mechanically ventilated mice. The measurements were taken three days after the last sensitization with HDM or saline in both WT and *ISM1*^*−/−*^ mice. Airway resistance in both HDM-sensitized WT and *ISM1*^*−/−*^ mice was markedly increased in response to methacholine challenge compared with their respective saline controls (Fig. 3E). Importantly, HDM-challenged *ISM1*^*−/−*^ mice showed a significant enhancement in airway resistance compared with WT mice, with significant differences observed at 5 mg/mL and 10 mg/mL methacholine exposure. On the other hand, the dynamic compliance in WT and *ISM1*^*−/−*^ mice was reduced with increasing methacholine concentration (Additional file 4: Fig. S3E), although the impact of ISM1 on dynamic compliance was not as significant as that on airway resistance. The increased basal level AHR in *ISM1*^*−/−*^ mice (saline control) correlates with structural alterations of the trachea in these mice. The tracheas in *ISM1*^*−/−*^ mice were significantly shorter and exhibited abnormal cartilage ring organization and reduced alpha-smooth muscle area, while other cell types such as ciliated epithelial cells, club cells, and goblet cells were not altered (Additional file 5: Fig. S4, A-F). These data indicate that ISM1 plays an important role in the structural integrity of the trachea and in restraining the development of AHR. Moreover, HDM-challenged *ISM1*^*−/−*^ mice presented more heightened AHR to methacholine above saline levels than WT mice, indicating enhanced airway inflammation. These results are consistent with the anti-inflammatory role of ISM1 in allergic asthma, as ISM1 deficiency led to increased airway inflammation and hyperresponsiveness.

**Fig. 4:**
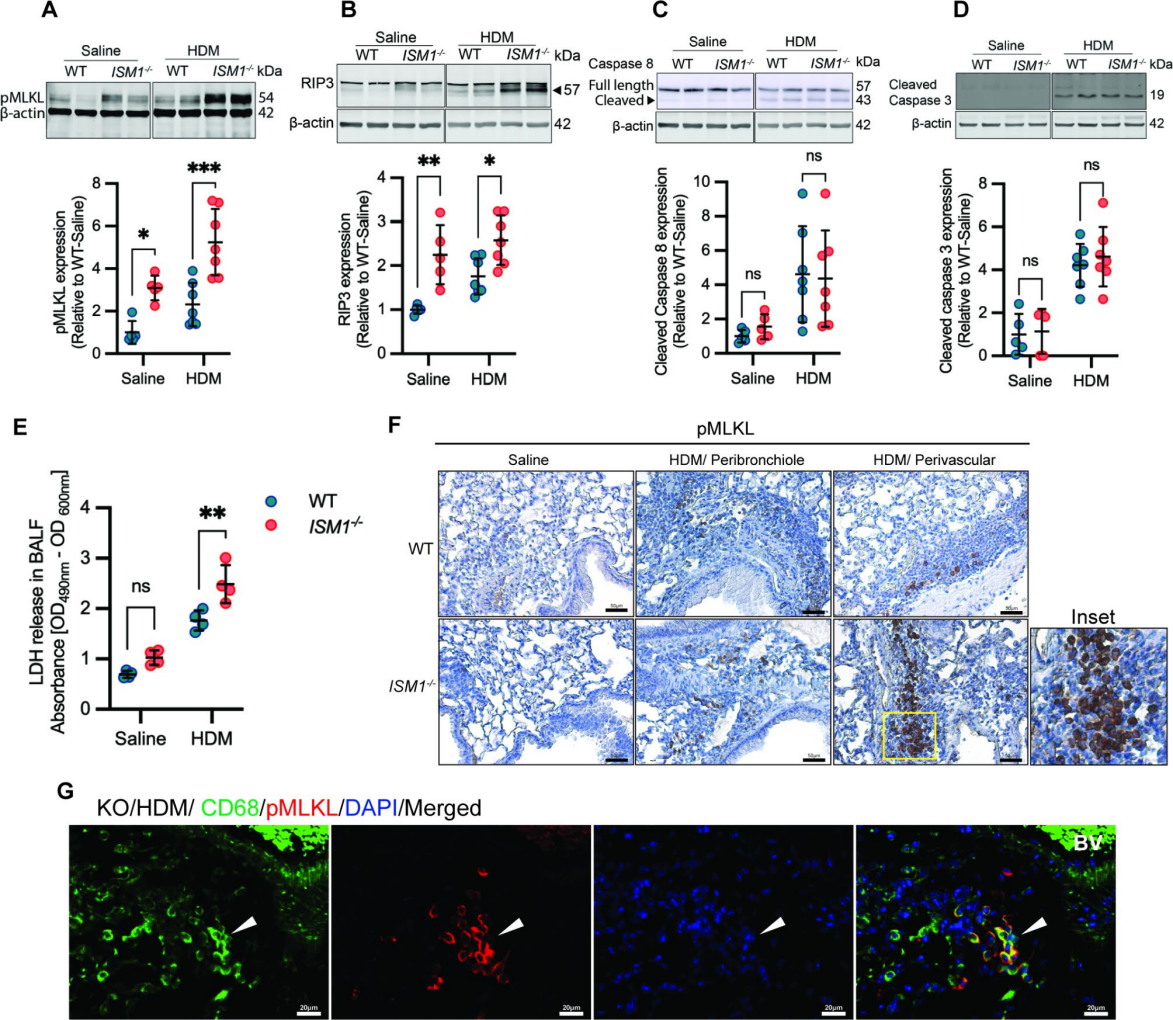
ISM1 regulates necroptosis in the lungs. **(A-D)** Representative Western blot analysis of **(A)** pMLKL, **(B)** RIP3, **(C)** cleaved caspase 8, and **(D)** cleaved caspase 3 protein expression in lung tissues comparing WT (n = 5) and *ISM1*^*−/−*^ (n = 7) mice with and without HDM sensitization. Data are presented as the mean ± SD. *P < 0.05; **P < 0.01; ***P < 0.001, using one-way ANOVA. **(E)** Release of pan-necrotic marker LDH in the BALF (n = 4). **(F)** Representative photomicrograph of immunohistochemical staining of lung tissue sections against pMLKL. Bars = 50 μm. **(G)** Immunofluorescence staining of lung sections from HDM-challenged *ISM1*^*−/−*^ mice. Bars = 20 μm. (*White arrows*) Indicates colocalization between pMLKL and the AM marker CD68. **(H)** rISM1 regulates pMLKL expression in MH-S cells. Data are presented as the mean ± SD. *P < 0.05; **P < 0.01; ****P < 0.0001, using one-way ANOVA. pMLKL, phosphorylated mixed lineage kinase domain-like protein; RIP, receptor-interacting protein 3; LDH, lactate dehydrogenase, Nec1, necrostatin 1

### ISM1 deficiency led to heightened lung inflammation and necroptosis

We next explored how ISM1 deficiency could lead to higher inflammation. Previously, we reported that ISM1 deficiency reduced AM apoptosis in the lungs with a concomitant increase in TNFα and inflammation [13]. Necroptosis is the lytic form of regulated cell death that can trigger inflammation. Therefore, we examined whether HDM extract could trigger a higher level of necroptosis in *ISM1*^*−/−*^ lungs, contributing to enhanced lung inflammation.

Western blot analysis revealed a significant increase in the expression of necroptosis markers, such as phosphorylated mixed lineage kinase domain-like protein (pMLKL) and receptor-interacting serine/threonine-protein kinase 3 (RIPK3), in the lungs of *ISM1*^*−/−*^ mice under basal (saline-treated) and HDM-treatment conditions. In particular, the levels of pMLKL and RIPK3 were significantly higher in *ISM1*^*−/−*^ lungs under HDM challenge than in the WT lungs (Fig. 4, A, B). However, there was no difference in caspase-8 and cleaved caspase-3 levels between *ISM1*^*−/−*^ and WT lungs under HDM challenge (Fig. 4, C, D). Lactate dehydrogenase (LDH) levels in the BALF of HDM-challenged *ISM1*^*−/−*^ mice were also significantly increased, supporting heightened necrosis (Fig. 4E). Immunohistochemical staining showed increased pMLKL^+^ macrophage-like cells in peribronchial and perivascular regions of HDM-challenged *ISM1*^*−/−*^ lungs (Fig. 4F). Consistently, immunofluorescence staining demonstrated colocalization of pMLKL with some CD68^+^ macrophages but not with AQ5^+^ (AT1) and SP-C^+^ (AT2) alveolar epithelial cells (Fig. 4G and Additional file 6: Fig. S5). These findings suggest that ISM1 deficiency led to heightened necroptosis in the lungs under HDM challenge, particularly in macrophage-like cells.

**Fig. 5:**
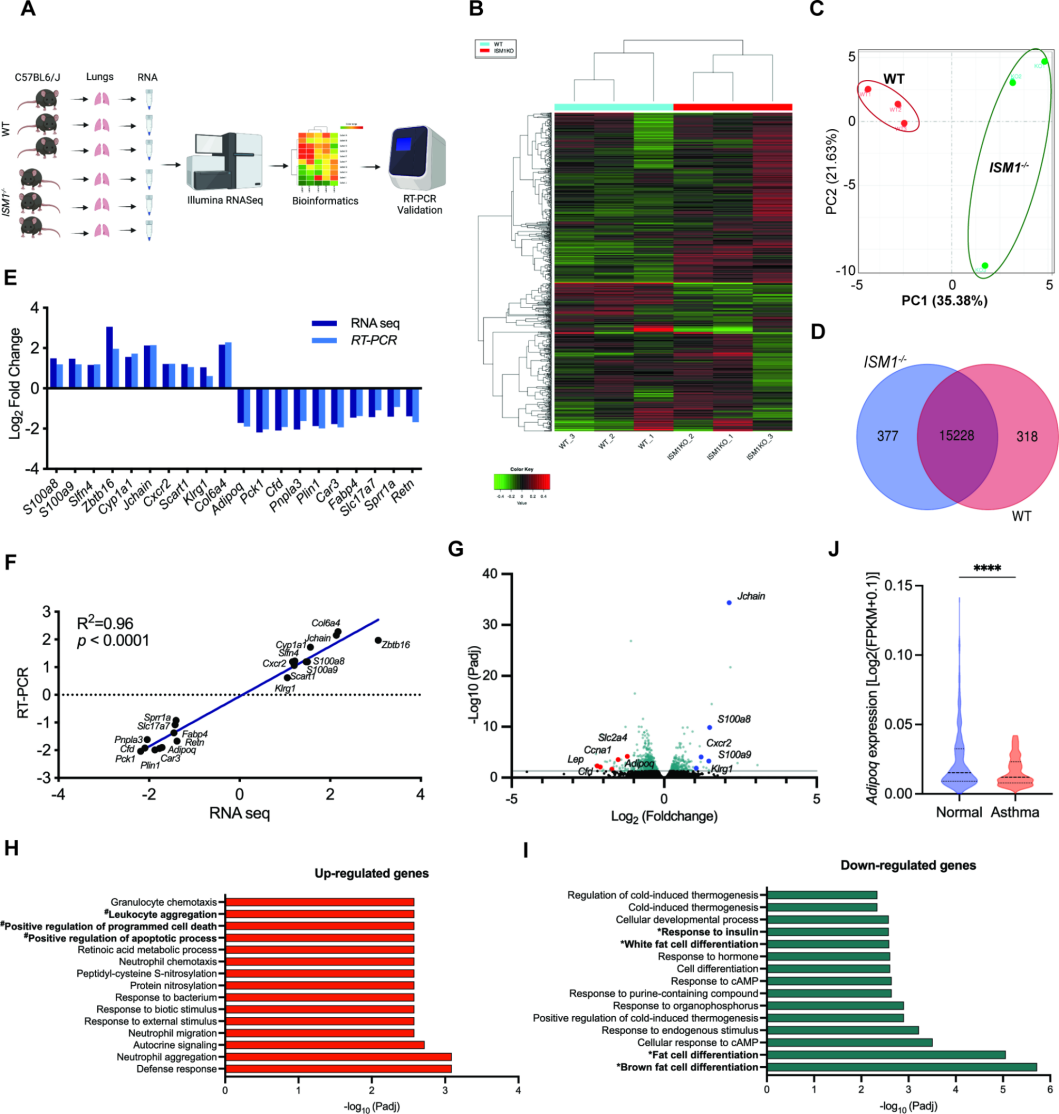
RNASeq analysis comparing WT and *ISM1*^*−/−*^ lungs at 2 months old. Immune-related gene set enriched in *ISM1*^*−/−*^ lungs suggests that *ISM1*^*−/−*^ mice are more prone to allergen-triggered immune responses. **(A)** Illustration of the RNASeq protocol. Total RNA was isolated from both sides of the lung tissue. **(B)** Unsupervised hierarchical clustering. **(C)** Principal component analysis of gene expression shows a distinction between *ISM1*^*−/−*^ samples from the WT. **(D)** Venn diagram presenting the number of genes that are unique or co-expressed in WT and *ISM1*^*−/−*^. **(E)** Validation of selected DE genes by qPCR. **(F)** Correlation between RNA-Seq and RT-PCR gene expression (R^2^ = 0.96, *p* < 0.0001). **(G)** The volcano plot illustrates the differentially expressed genes for each comparison between WT and *ISM1*^*−/−*^ lungs. A cutoff of 0.05 adjusted P value and twofold changes were used. Some significant DE genes were annotated. **(H)** Enriched GO biological processes of up-regulated genes. **(I)** Enriched GO biological processes of down-regulated genes. All the terms shown are enriched in *ISM1*^*−/−*^ lungs. **(J)***Adipoq* expression comparing normal and asthmatic human lungs. RNA-Seq data were obtained and analyzed from the public databases Genotype-Tissue Expression B37 and B38_GC33 projects using Ingenuity Pathway Analysis software (OmicsSoft, Qiagen)

### RNA-Seq analysis revealed an altered transcriptome in *ISM1*^*−/−*^ lungs

We conducted a comparative transcriptomic analysis of WT and *ISM1*^*−/−*^ mouse lung tissue at baseline to understand the mechanism of ISM1’s anti-inflammatory function in asthma (Fig. 5A). Using iDEP.951 software, we filtered out lowly expressed genes (minimum count per million = 0.5 in at least 1 sample) from 54,532 genes in 6 samples, resulting in 18,689 genes passing the filter. We then transformed the read count data using the regularized logarithm (rlog) method for clustering analysis and principal component analysis (PCA). The hierarchical clustering of the top 1000 genes ranked by their standard deviation across all samples showed minimal variation among the biological replicates with each group (Fig. 5B). The PCA plot also confirmed a distinguishable difference between the *ISM1*^*−/−*^ and the WT samples (Fig. 5C). Co-expression analysis revealed 377 genes uniquely expressed in *ISM1*^*−/−*^ lungs and 318 genes uniquely expressed in WT lungs (Fig. 5D). With the DESeq2 R package, we identified 48 up-regulated and 67 down-regulated genes whose expression changed at least 2-fold in *ISM1*^*−/−*^ lungs using a threshold of false discovery rate (FDR) < 0.1 (additional file 1: Table S3). The expression of selected differentially expressed genes was validated with quantitative PCR (RT-PCR) (R^2^ = 0.96, p < 0.0001) (Fig. 5, E, F). The volcano plot illustrated the distribution of validated DE genes for each comparison between WT and *ISM1*^*−/−*^ lungs (Fig. 5G).

We conducted Gene Ontology (GO) and KEGG pathway analyses to understand the significance of our findings. The upregulated genes in *ISM1*^*−/−*^ mice were associated with the GO regulation of innate immune systems and response to external stimuli (Fig. 5H and Additional file 1: Table S4), indicating airway hypersensitivity. In contrast, the downregulated genes were enriched in the GO cellular developmental process, fat cell differentiation, and response to endogenous stimulus (Fig. 5I and Additional file 1: Table S5). In addition, the PPAR signaling pathway, type II diabetes, adipocytokine signaling pathway, and AMPK signaling pathway were also enriched in the downregulated genes (Table 1). These findings align with a recent report that identified ISM1 as an adipokine [36]. Notably, ISM1 deficiency led to a significant downregulation of adiponectin (*Adipoq*) in mouse lungs, and Ingenuity Pathway Analysis of human RNA-Seq data from public databases Genotype-Tissue Expression B37 and B38_GC33 projects also confirmed that adiponectin expression is reduced in human asthmatic lungs (Fig. 5J and Additional file 1: Table S6).

Adiponectin is a protein hormone mainly secreted by adipocytes that regulates lipid and glucose metabolism [37]. It also possesses anti-inflammatory properties and has been demonstrated to attenuate allergen-induced airway inflammation and hyperresponsiveness in mice [18, 19]. Notably, *Adipoq*^−/−^ mice also present spontaneous emphysema phenotype similar to *ISM1*^*−/−*^ mice in early adulthood [38]. Despite its potential therapeutic benefits, the precise role of adiponectin in human asthma has not been consistent [39]. Our findings here suggest that ISM1 may exert its anti-inflammatory function in allergic-like airway/lung inflammation via adiponectin regulation.

**Table 1:** Enriched KEGG pathways in up-regulated and down-regulated genes

Pathways	P_adj_	Genes	Regulated direction
PPAR signaling pathway	1.63E-7	*Plin4 Rxrg Adipoq Pck1 Plin1 Ucp1 Fabp4*	Down
Type II diabetes mellitus	0.0003	*Slc2a4 Adipoq Abcc8 Kcnj11*	Down
Adipocytokine signaling pathway	0.0010	*Rxrg Slc2a4 Adipoq Pck1*	Down
AMPK signaling pathway	0.0070	*Slc2a4 Adipoq Pck1 Ccna1*	Down
IL-17 signaling pathway	0.0072	*S100a8 S100a9*	Up

### ISM1 stimulates adiponectin expression in lung AT2 cells

**Fig. 6:**
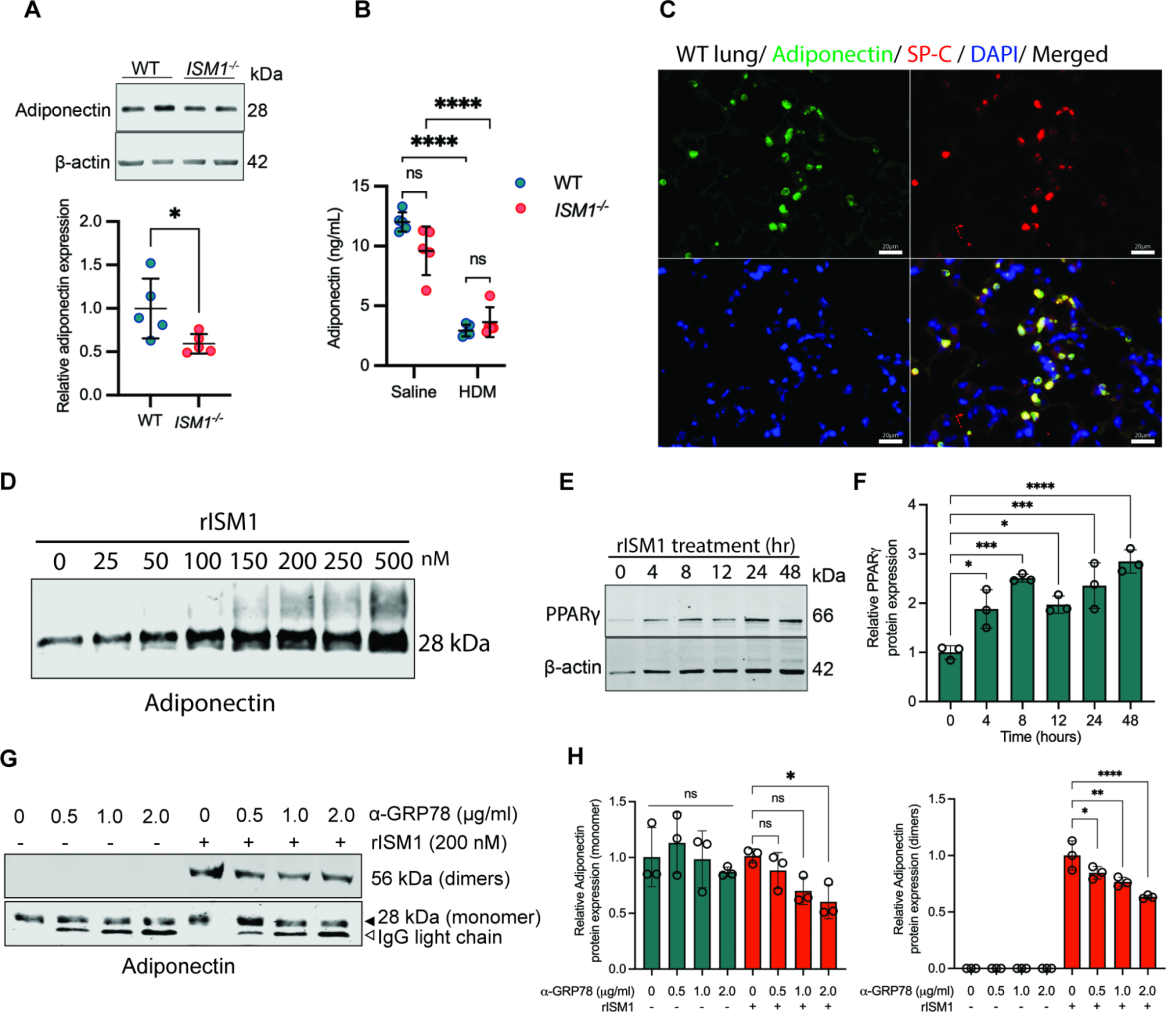
ISM1 regulates adiponectin expression in the lungs. **(A)** Relative adiponectin protein expression in lung lysates (n = 5). Data are presented as the mean ± SD. *P < 0.05, using Student’s t test. **(B)** Adiponectin level in the BALF of HDM-challenged mice compared with the saline control measured with ELISA (n = 5). Data are presented as the mean ± SD. ****P < 0.0001, using two-way ANOVA. **(C)** Immunofluorescence images showing colocalization between adiponectin and SP-C in WT lungs. Bars = 20 μm. **(D)** Adiponectin expression in conditioned media from A549 cells treated with different doses of rISM1. **(E-F)** PPARγ protein expression and quantification in A549 cell lysates post-treated with 200 nM of rISM1 measured at different time points. **(G-H)** Adiponectin expression and quantification in A549 conditioned media under 200 nM rISM1 with or without GRP78 antibody neutralization. Data are presented as the mean ± SD. *P < 0.05; **P < 0.01; ***P < 0.001; ****P < 0.0001, using one-way ANOVA. SP-C, surfactant protein C; PPARγ, peroxisome proliferator-activated receptor gamma; GRP78, glucose-regulating protein 78

To investigate the potential influence of ISM1 on adiponectin expression, we conducted a comparison of adiponectin protein levels in lung lysates between *ISM1*^*−/−*^ mice and WT mice at the basal level. Indeed, our results indicate that ISM1 deficiency caused a significant 50% reduction in adiponectin expression in the lungs (Fig. 6A). Furthermore, in HDM-induced allergic-like airway/lung inflammation, where ISM1 is downregulated, we observed a significant reduction in adiponectin levels in BALF from both WT and *ISM1*^*−/−*^ mice (Fig. 6B). To determine the source of adiponectin secretion in the lungs, we performed immunofluorescence staining and found that adiponectin-positive cells colocalized with the SP-C marker, indicating that airway epithelial type 2 (AT2) cells are responsible for secreting adiponectin in the lungs (Fig. 6C). Additionally, we confirmed that adiponectin expression was not correlated with CD68 expression (Additional file 7: Fig. S6A), indicating that AMs are not the source of adiponectin. Moreover, our findings suggest that the regulatory role of ISM1 on adiponectin expression is specific to the lungs, as we observed no significant alterations in adiponectin expression in adipose tissues obtained from iWAT, gWAT, and BAT between WT and *ISM1*^*−/−*^ mice (Additional file 7: Fig. S6B).

To confirm that ISM1 plays a role in stimulating adiponectin expression, we treated A549 cells (human AT2 cell line) with different doses of rISM1 and measured the release of adiponectin in the conditioned media. Our results revealed that adiponectin secretion in A549 cells was stimulated in a dose-dependent manner by ISM1 (Fig. 6D). Furthermore, we observed an increase in the expression of PPARγ, a key transcription factor for adiponectin, in A549 cells as early as 4 h post-treatment with rISM1 (Fig. 6, E, F) [40]. Previously, we identified two cell-surface receptors for ISM1, the high-affinity receptor cell surface GRP78 (csGRP78) and the low-affinity receptor integrin αvβ5 [11, 41]. Using antibody neutralization experiments, we demonstrated that blocking csGRP78 but not integrin αvβ5 reduces adiponectin secretion in A549 cells (Fig. 6, G, H and Additional file 7: Fig. S6C). Our findings suggest that ISM1 stimulates adiponectin expression in lung AT2 cells by partially functioning through csGRP78 and involving downstream PPARγ signaling pathways. Overall, our results provide insights into the mechanism by which ISM1 regulates adiponectin expression in the lungs.

### Intratracheal delivery of rISM1 suppresses allergic Asthma inflammation in mice

**Fig. 7:**
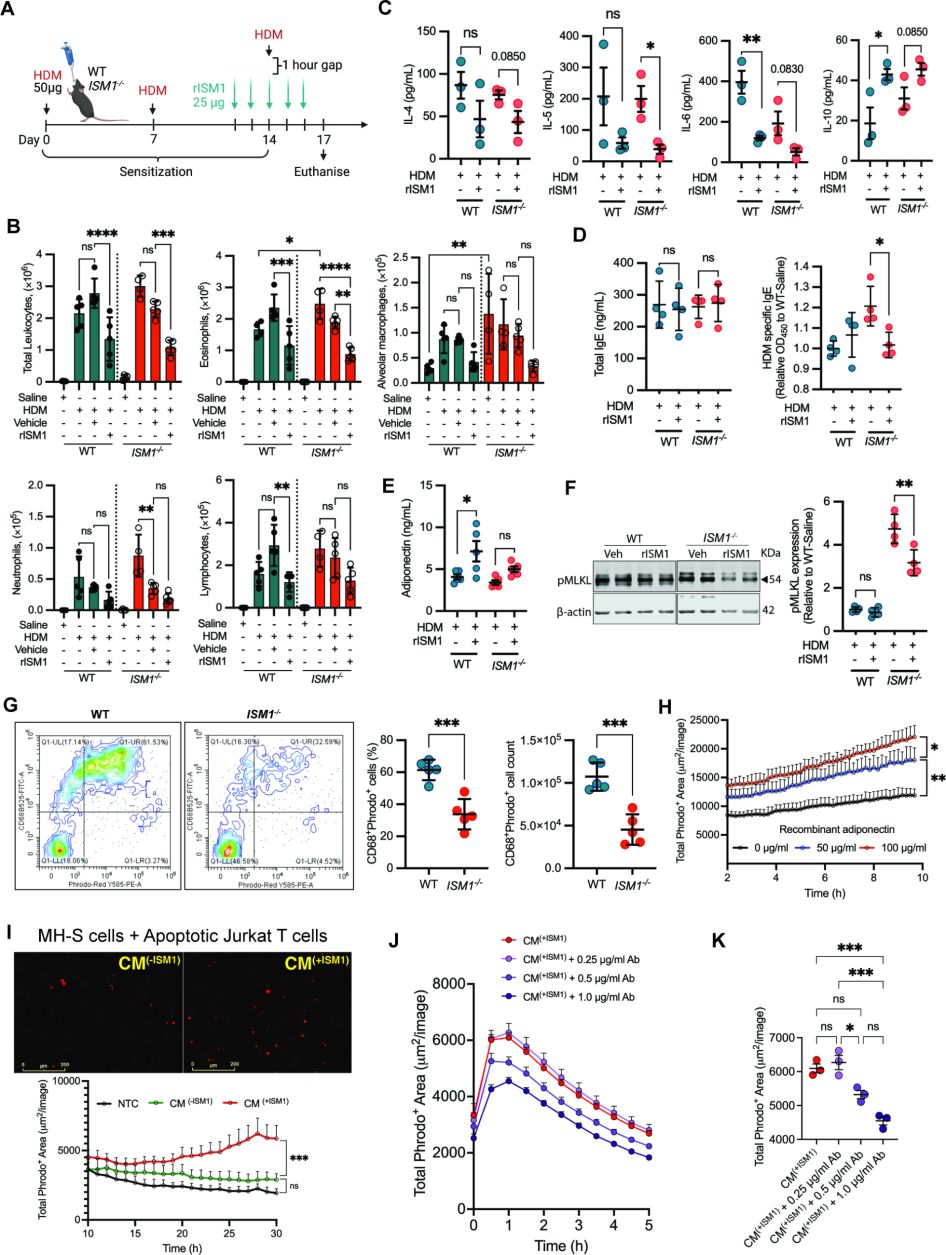
Asthma exacerbation is alleviated upon exogenous recombinant ISM1 supplementation. **(A)** Illustration of the HDM sensitization and rISM1 supplementation protocols. **(B)** Flow cytometry analysis of the immune cell composition in BALF comparing sensitized and non-sensitized controls (n = 4), vehicle-treated, and rISM1-treated groups (n = 5). **(C)** The amount of IL4, IL5, IL6, and IL10 cytokines in BALF (n = 3). **(D)** Serum titer of total and HDM-specific IgE comparing WT and *ISM1*^*−/−*^ mice with or without rISM1 supplementation (n = 4). **(E)** Adiponectin levels in BALF (n = 5). **(F)** Representative western blot analysis of pMLKL expression in lung tissues post-rISM1 treatment (n = 4). **(G)** Efferocytosis of UV-irradiated Jurkat T cells by alveolar macrophages in vivo (n = 5). The CD68-Phrodo red double positive population was efferocytotic AMs. Data are presented as the mean ± SD. ***P < 0.001, using Student’s t test. **(H)** Recombinant adiponectin enhanced MH-S efferocytosis of UV-irradiated Jurkat T cells measured using Phrodo-red dye. **(I)** rISM1-induced adiponectin in conditioned media (CM) promotes efferocytosis of UV-irradiated Jurkat T cells by MH-S cells. **(J)** The effect of antibody neutralization of ISM1-induced adiponectin on MH-S efferocytosis. The experiment was repeated three times independently. **(K)** Total Phrodo^+^ area quantified at 1 h after apoptotic Jurkat T cells were introduced to MH-S cells. Data are presented as the mean ± SD. *P < 0.05; **P < 0.01; ***P < 0.001; ****P < 0.0001, using one-way ANOVA.

To determine whether airway delivery of exogenous rISM1 could have a therapeutic effect on allergic asthma inflammation, we expressed and purified mouse rISM1 from CHO cells. Our purified 70 kDa rISM1 was shown to be proapoptotic on thapsigargin-pretreated MH-S cells, consistent with our earlier report using *E. coli*-derived rISM1 (Additional file 8: Fig. S7, A-C) [13]. We then intratracheally administered either CHO-derived rISM1 or vehicle to both *ISM1*^*−/−*^ and WT mice in the HDM-induced allergic asthma model (Fig. 7A). Our results showed that airway administration of rISM1 significantly reduced HDM-induced total leukocyte and eosinophil numbers in BALF compared to the vehicle control. Although HDM-induced allergic-like airway/lung inflammation presented increased neutrophils and lymphocytes in both WT and *ISM1*^*−/−*^ lungs, it did not further increase AMs in *ISM1*^*−/−*^ mice, which already had a higher basal level without HDM [13]. Nevertheless, airway delivery of rISM1 resulted in a trend of reduction in all these cell types in both WT and *ISM1*^*−/−*^ mice, even though some of the reductions were not statistically significant (Fig. 7B).

Furthermore, the levels of IL-4, IL-5, and IL-6 all showed a trend of reduction in BALF in both WT and *ISM1*^*−/−*^ lungs after rISM1 administration, albeit the changes in IL-4 were not statistically significant. In contrast, IL-10 showed a trend of upregulation in the rISM1-treated groups (Fig. 7C). The BALF levels of TNFα and IL-21, which were significantly higher in *ISM1*^*−/−*^ mice upon HDM challenge (Fig. 3, B, C), did not show a difference post-rISM1 treatment (Additional file 9: Fig. S8A). In addition, a significant reduction in HDM-specific IgE but not total IgE levels was observed upon rISM1 treatment (Fig. 7D). Importantly, BALF adiponectin levels were significantly increased in the WT lungs after rISM1 treatment, while a mild increase was also observed in *ISM1*^*−/−*^ lungs (Fig. 7E). Moreover, the necroptotic marker pMLKL was significantly reduced in *ISM1*^*−/−*^ lungs after rISM1 treatment (Fig. 7F). Altogether, these data indicate that airway-delivered exogenous rISM1 can effectively suppress inflammation in HDM-triggered allergic asthma.

HDM-induced allergic asthma in mice is a well-established model closely resembling the eosinophil-dominant Th2 type of allergic asthma seen in humans [42, 43]. Eosinophils have a short lifespan of about 3–4 days before they undergo apoptosis and are cleared by AMs [44, 45]. Delayed clearance of apoptotic cells could lead to secondary necrosis and further release of pro-inflammatory cytokines that could accelerate inflammation [45]. Macrophages are primarily responsible for clearing apoptotic cells through efferocytosis, which plays an important role in inflammation resolution [45, 46]. Adiponectin has been shown to enhance macrophage efferocytosis by opsonizing early apoptotic cells by binding to membrane phospholipids [20, 47]. Given that *ISM1*^*−/−*^ lungs have reduced adiponectin levels, it is reasonable to suggest that low adiponectin may delay the clearance of dead eosinophils by AMs, leading to increased necroptosis and the release of more proinflammatory cytokines, resulting in more severe inflammation.

To investigate whether ISM1 promotes macrophage efferocytosis by stimulating adiponectin production in vivo, we instilled UV-irradiated early apoptotic Jurkat T cells labeled with pHrodo-red into the mouse airway via intratracheal delivery. BAL cells were collected 90 min after the mouse had awakened from anesthesia. Co-expression of the pHrodo-red signal on CD68^+^ cells in the BALF was analyzed using flow cytometry. In the BALF, the majority of CD68^hi^ cells representing alveolar macrophages also expressed CD11c and Siglec F [48, 49]. Although monocytes and dendritic cells also express CD68, our data showed that HDM sensitization did not increase BALF macrophage number (Fig. 2). Hence, the contribution of monocytes in this disease model is negligible. In addition, the number of dendritic cells in the BALF is also known to be very low [50, 51]. Thus, most likely the increased pHrodo-red dye signal in the acidic pH environment of phagosomes of AMs indicates the engulfment of apoptotic cells by AMs, i.e., efferocytosis. Our results showed that *ISM1*^*−/−*^ lungs had significantly reduced CD68^+^pHrodo-red^+^ cells compared to WT lungs, indicating that ISM1 deficiency hampered AM efferocytosis of apoptotic cells (Fig. 7G and Additional file 9: Fig. S8B).

To confirm whether the reduced AM efferocytosis under ISM1 deficiency is due to reduced adiponectin in *ISM1*^*−/−*^ lungs, we pre-incubated apoptotic Jurkat cells with recombinant adiponectin before mixing them with mouse MH-S AMs in vitro. Adiponectin dose-dependently enhanced AM phagocytosis (Fig. 7H). Furthermore, to demonstrate that ISM1-induced adiponectin production enhances efferocytosis, we pre-incubated apoptotic Jurkat T cells with conditioned media (CM) from ISM1-treated A549 cells (CM^(+ISM1)^) or control A549 cells (CM^(−ISM1)^). Our results showed that CM^(+ISM1)^ but not CM^(−ISM1)^ significantly enhanced AM efferocytosis (Fig. 7I and Additional file 9: Fig. S8C, D). In contrast, antibody neutralization of adiponectin secreted in CM^(+ISM1)^ significantly reduced the capacity of MH-S cell efferocytosis in a dose-dependent manner (Fig. 7, J, K). These findings support the notion that ISM1 enhances AM efferocytosis by stimulating lung adiponectin production, facilitating the rapid clearance of apoptotic cells in the allergic airway, and hence prohibiting secondary necroptosis, which can cause further inflammation.

## Discussion

We demonstrated here for the first time the role of ISM1 in regulating airway inflammation in response to aeroallergens. ISM1 deficiency exacerbated HDM-induced AHR in mice, while intratracheal administration of rISM1 suppressed HDM-induced allergic-like airway/lung inflammation (Figs. 1, 2, 3 and 7). Moreover, we showed that *ISM1*^*−/−*^ lungs harbored significantly lower adiponectin, while rISM1 dose-dependently stimulated adiponectin secretion in cultured AT2 alveolar epithelial cells (Figs. 5 and 6). We demonstrated that ISM1-induced adiponectin acts as an opsonizing agent in enhancing AM efferocytosis of apoptotic cells both in vitro and in vivo. Reduced AM efferocytosis under ISM1 deficiency likely led to increased secondary necroptosis, resulting in more severe inflammation in *ISM1*^*−/−*^ mice in HDM-induced allergic asthma. Indeed, necroptosis in *ISM1*^*−/−*^ lungs under HDM challenge was significantly elevated, as demonstrated by upregulated pMLKL and RIP3 levels in lung lysates and increased LDH release in BALF. Immunofluorescence staining further revealed that at least some AMs were undergoing necroptosis. Overall, our findings reveal a novel anti-inflammatory mechanism in airways mediated through the ISM1-adiponectin signaling axis.

Importantly, we found that *Ism1* mRNA is consistently downregulated in allergic airways from both mice and humans through in silico analysis (Fig. 1). This observation aligns with the work of Roffel et al., who identified higher expression of miR-223-3p in asthma patients. This miRNA was predicted to negatively regulate ISM1 expression (Supplementary Table S3) [52]. The downregulation of ISM1 expression upon HDM sensitization shown by qRT-PCR in the present study validated the RNA sequencing data. Interestingly, in contrast to asthma conditions, our earlier study found that ISM1 expression was upregulated in an LPS-induced acute lung injury mouse model [53]. Similarly, ISM1 levels increased in the mouse bronchial epithelium following cigarette smoke exposure [13]. Notably, both LPS and cigarette smoke induce a T_H_1-biased inflammatory response primarily through the activation of TLR-4 and downstream NFκB signaling pathways. A recent study by Rivera-Torruco et al. reported an increase in ISM1^+^ lung hematopoietic progenitor cells during *P. aeruginosa* infection in mice, which is also a T_H_1-type inflammatory response [54]. In contrast, HDM sensitization is associated with a T_H_2-type inflammatory response. These data together suggest that different inflammatory disease models may impact ISM1 expression or ISM1 + cells differently.

We wish to point out that there was no significant increase in immune cell numbers in *ISM1*^*−/−*^ mice under saline challenge (Fig. 2C), although a less pronounced version of this phenotype was observed in a different set of experiments (Fig. 7B). In our initial observations, we reported that naïve *ISM1*^*−/−*^ lungs presented spontaneous low-grade inflammation with higher leukocyte numbers than WT lungs [8]. In addition to the possibility that saline instillation into the airway triggers a certain level of immune response, we would like to emphasize that the phenotype of higher leukocyte number appeared to be weaker in *ISM1*^*−/−*^ mice generated from the C57BL/6J background than in those generated from the FVB/N background [13]. Additionally, it is crucial to acknowledge that phenotypes can diminish over time due to possible adaptation through prolonged and repeated breeding in mice. Nevertheless, we demonstrated that ISM1-deficient mice displayed an enhanced inflammatory response following HDM challenge (Fig. 2). These results align with our earlier report indicating that ISM1 deficiency intensified acute lung inflammation in response to LPS challenge, and the inflammatory phenotypes were reversed by exogenous supplementation of rISM1 [8]. Collectively, these findings suggest that ISM1 may be critical in limiting airway inflammation in asthma.

Compared with WT mice, *ISM1*^*−/−*^ mice exhibited higher levels of serum IgE upon HDM challenge but similar levels of IL-4, IL-5, and IL-13 (Fig. 2B A). These discrepancies could be due to the T_H_1 leaning immune responses of the C57BL/6 strain as compared to BALB/c, a more commonly used strain for asthma studies with T_H_2-prone immune responses [55, 56]. In addition, the time of cytokine detection could also be a contributing factor. It has been shown that T_H_2 cytokines peak at 2 weeks post-HDM challenge and subside from 3 weeks onward, although AHR persists [57]. Furthermore, the production and regulation of IgE antibodies involve multiple factors and complex interactions involving various cytokines and cellular signaling pathways. According to a previous study, while IgE synthesis exhibited a positive correlation with the concentration of IL-4 and IL-13, a robust inverse correlation was also observed between the amount of IgE produced and the concentration of IFNγ, and a potent IgE response can be induced with relatively low levels of IL-4 and IL-13, provided that the levels of IFNγ are also low [58]. Our results align with these findings, where the IFNγ level was significantly lower in HDM-sensitized *ISM1*^*−/−*^ mice (Fig. 3B). Moreover, TGFβ_1_, whose cleaved active form was found to be highly expressed in *ISM1*^*−/−*^ mice upon HDM challenge, could also be a potential contributing factor (Fig. 2I). TGFβ has been demonstrated to inhibit T_H_2 development by suppressing IL-4 production [59]. Meanwhile, IL-21 is also known to be a negative regulator of IgE class switch recombination in the geminal center [60]. A higher level of IL-21 in BALF under HDM challenge in *ISM1*^*−/−*^ mice, while serum IgE is higher, suggests that other regulatory factors may also be involved. Nevertheless, further investigation is necessary to understand the specific role that ISM1 plays in regulating the adaptive immune response.

Adiponectin is an anti-inflammatory adipokine mainly secreted in adipocytes but is also expressed in the lungs. Studies have shown that low adiponectin levels are associated with type 2 diabetes and obesity [61, 62]. Obesity is an important comorbidity in asthmatic patients [63]. In obese-asthma patients, macrophage efferocytosis was 40% lower than that of non-obese subjects [64]. Moreover, macrophages derived from diet-induced obese mice presented impaired efferocytosis [65, 66]. Similarly, macrophages from diabetic mice also showed significant impairment in efferocytosis [67]. Furthermore, obese HDM mice have been reported to present reduced plasma adiponectin and higher macrophage infiltration into the lungs and BALF [68]. ISM1 was recently identified as an adipokine secreted by adipocytes, that promotes glucose uptake in an insulin-like fashion [36]. However, contrary to *ISM1*^*−/−*^ lungs, adiponectin expression was shown to have no significant alteration in *ISM1*^*−/−*^ adipocytes [36]. We demonstrate here that *Adipoq* mRNA, which encodes for the adiponectin protein, was significantly downregulated in *ISM1*^*−/−*^ lungs (Figs. 5 and 6). ISM1 dose-dependently stimulated adiponectin secretion in lung AT2 cells and promoted AM efferocytosis (Fig. 7). Consistently, adiponectin-deficient mice reported an impaired clearance of apoptotic cells, while adiponectin supplementation showed a reversal effect [23]. Adiponectin-deficient mice presented heightened allergic airway inflammation and spontaneous emphysema, phenotypes similar to those of ISM1-deficient mice [19, 38]. Systemic adiponectin application has been reported to attenuate allergen-induced airway inflammation and hyperresponsiveness in mice [18]. Hence, ISM1 may suppress allergic airway inflammation by stimulating lung adiponectin production and promoting adiponectin-mediated AM efferocytosis. Note that our previous report suggested no obvious alteration in efferocytosis of AMs obtained from *ISM1*^*−/−*^ mice in high-serum media [13]. We speculate that the high serum culture media used might have masked the efferocytosis deficiency since a high amount of adiponectin is known to be present in serum [69].

In eosinophil-dominant type-2 allergic asthma, apoptotic eosinophils are cleared by AMs through efferocytosis [70]. Clearance of apoptotic cells by lung AMs has been reported to prevent the development of HDM-induced allergic airway inflammation [45]. It has also been reported that efferocytosis is impaired in non-eosinophilic asthma [71]. Delayed clearance of apoptotic cells in *ISM1*^*−/−*^ mice could trigger secondary necroptosis, leading to heightened airway inflammation. Meanwhile, our RNASeq also identified that genes associated with damage-associated molecular patterns (DAMPs) such as S100a8 and S100a9, were highly upregulated (Table 1). Upregulation of DAMPs also suggests the involvement of increased necroptosis in inflammation [72]. These findings together support a mechanism whereby ISM1 restrains airway inflammation and hyperresponsiveness by promoting adiponectin production in the airways. Nevertheless, the possibility that ISM1 also reduces eosinophil infiltration into the airways cannot be excluded.

Alveolar epithelial cells are composed of two major cell types, alveolar type 1 (AT1) and alveolar type 2 (AT2) cells, which cover 99% of the internal surface of the lungs. Besides functioning as a barrier separating the internal and external environments, epithelial cells are also immunologically active which sense changes in the airway environment and interact with immune cells [73]. AT2 cells are responsible for repairing processes and modulating immune responses upon lung injury [74]. AT2 cell apoptosis has been implicated in the pathogenesis of idiopathic pulmonary fibrosis [75]. In asthma, AT2 cells protect the lungs against allergen-induced airway inflammation by secreting TGFβ_1,_ which stimulates regulatory T cell (Treg) development [76]. The Treg-secreted anti-inflammatory cytokine IL-10 is involved in the suppression of T_H_17-mediated inflammation [77]. Indeed, lung tissue from *ISM1*^*−/−*^ mice produced significantly less IL-10 than that of WT mice under HDM sensitization. Furthermore, while HDM treatment markedly reduced BALF IL-21 in WT mice, ISM1 deficient mice maintained a relatively high level of BALF IL-21 post-HDM sensitization (Fig. 3). Previous studies have reported that IL-21 and its receptor are necessary for the development of eosinophilic airway inflammation [78, 79]. In human asthma, elevated IL-21 expression correlates with disease severity [79]. IL-21 has been reported to have an immunoregulatory function via its ability to induce IL-10 production [80] and directly induce Treg apoptosis [30]. Nevertheless, there was no increase in IL-21 in *ISM1*^*−/−*^ lungs after HDM challenge compared to saline treated *ISM1*^*−/−*^ lungs, hence IL-21 could not be responsible for the reduced IL-10 in *ISM1*^*−/−*^ lungs under HDM sensitization. How ISM1 deficiency leads to a reduction in lung IL-10 requires further investigation. These findings together suggest that ISM1 could exert its anti-inflammatory function in the airways by promoting the production of anti-inflammatory cytokines such as adiponectin and IL-10.

AMs are the major immune cells in the alveolar space, and they often adhere to the alveolar epithelium and play a predominantly immune-suppressive role in maintaining homeostasis. Lung epithelial cells are known to secrete anti-inflammatory proteins such as CD200 to maintain AM in a quiescent state and limit inflammatory amplitude [81]. In conjunction with our previous report that *ISM1*^*−/−*^ lungs harbor more activated pro-inflammatory AMs and trigger spontaneous emphysema [8, 13], our findings here showed that HDM challenge generated a more severe allergic airway inflammation with pre-existing emphysema. *ISM1*^*−/−*^ mice may thus be useful in studying asthma-COPD overlap syndrome, whose molecular mechanisms remain poorly studied [82, 83]. Future studies using lung-specific *ISM1*^*−/−*^ mouse lines would help to further clarify the roles of ISM1 in the lungs.

## Conclusions

In conclusion, this work reveals a novel role of ISM1 in curbing allergen-induced airway inflammation and hyperresponsiveness by stimulating airway expression of the anti-inflammatory adiponectin. ISM1 may have therapeutic potential for allergic asthma.

### Electronic supplementary material

Below is the link to the electronic supplementary material.

Supplementary Material 1

Supplementary Material 2

Supplementary Material 3

Supplementary Material 4

Supplementary Material 5

Supplementary Material 6

Supplementary Material 7

Supplementary Material 8

Supplementary Material 9

Supplementary Material 10

## Data Availability

All data generated or analyzed during this study are included in this published article and its supplementary information files. RNASeq data comparing *ISM1*^*−/−*^ and WT lungs at 2 months old are available from Gene Expression Omnibus (GEO) database (GSE215234).

## Abbreviations

AHR: Airway hyperresponsiveness
AM: Alveolar macrophages
APN: Adiponectin
AT2: Alveolar type 2 epithelial cells
BALF: Bronchoalveolar lavage fluid
csGRP78: Cell surface glucose regulated protein 78-kDa
DAMPs: Damage-associated molecular patterns
ER: Endoplasmic reticulum
GO: Gene ontology
MLKL: Mixed lineage kinase domain-like protein
RIP3: Receptor-interacting protein kinase 3
HDM: House dust mite
ISM1: Isthmin-1
*i.t.*: Intratracheally
IFN-γ: Interferon-gamma
*i.p.*: Intraperitoneally
MCP-1: Monocyte chemoattractant protein-1
NK: Natural killer
PPARγ: Peroxisome Proliferator-Activated Receptor Gamma
p-MLKL: Phosphorylated mixed lineage kinase domain-like protein
TBS: Tris buffer saline
TNF-α: Tumor necrosis factor-alpha
T_H_1: T helper type 1 cells
T_H_2: T helper type 2 cells
TGFβ_1_: Transforming growth factor beta 1
Tregs: Regulatory T cells
SABA: Short-acting beta-agonists
WT: Wild type
