# Supplementary figures and images for: Isthmin-1 attenuates allergic Asthma by stimulating adiponectin expression and alveolar macrophage efferocytosis in mice
Source: Respir Res. 2023 Nov 6;24:269. doi: 10.1186/s12931-023-02569-1 (PMC10626717; doi:10.1186/s12931-023-02569-1)

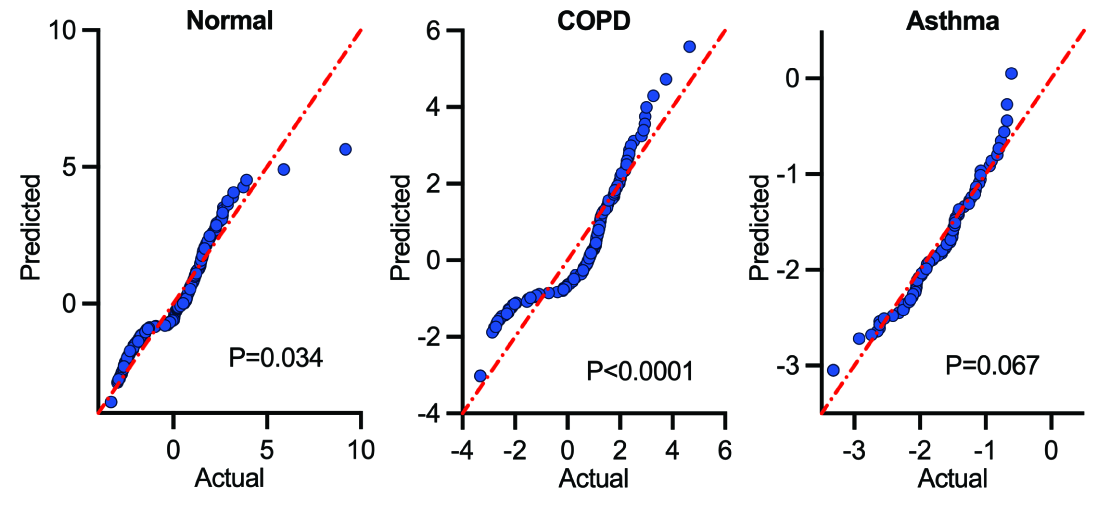

Supplement: Supplementary file 2 — Supplementary Material 2 [file 12931_2023_2569_MOESM2_ESM.tif]

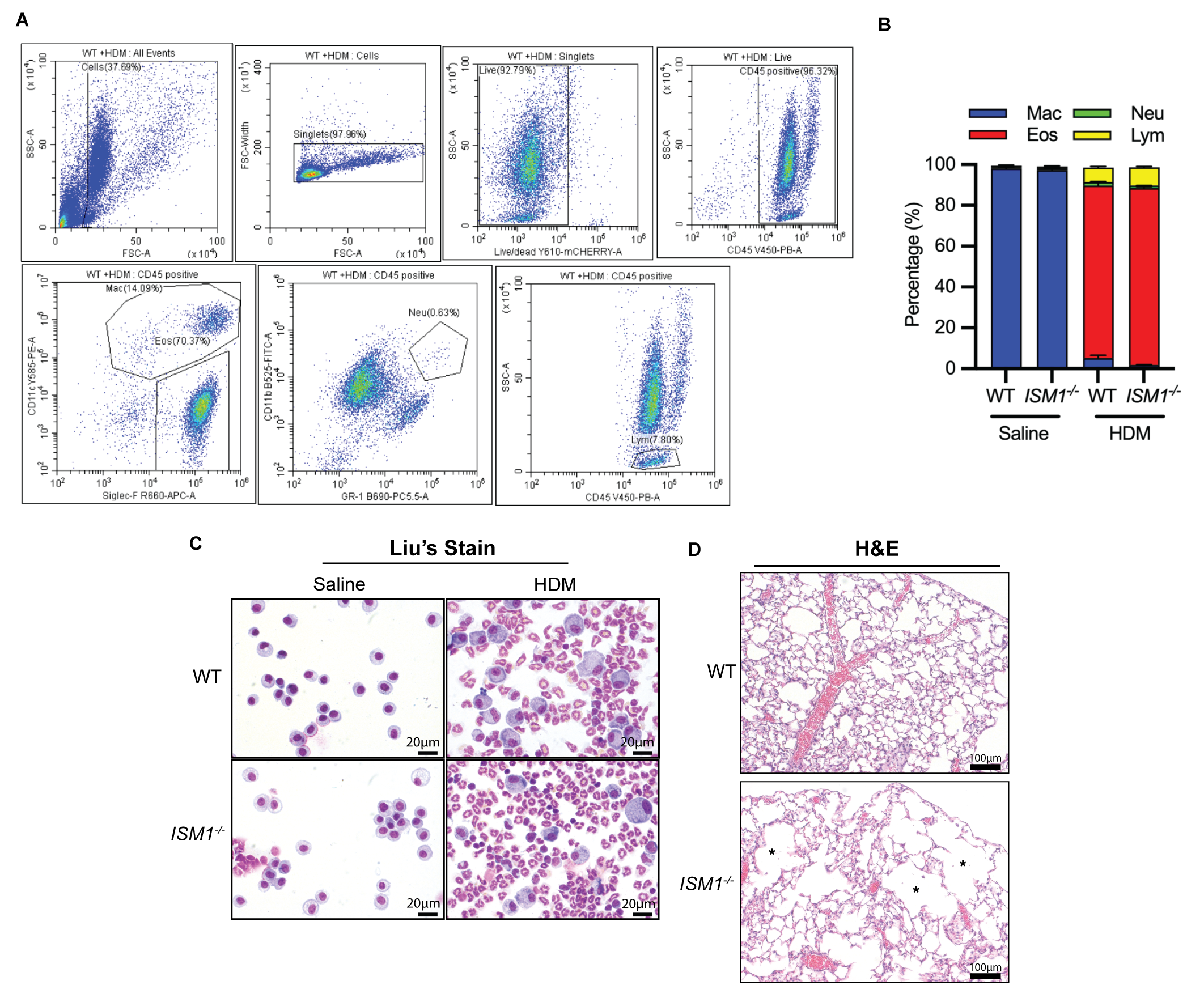

Supplement: Supplementary file 3 — Supplementary Material 3 [file 12931_2023_2569_MOESM3_ESM.tif]

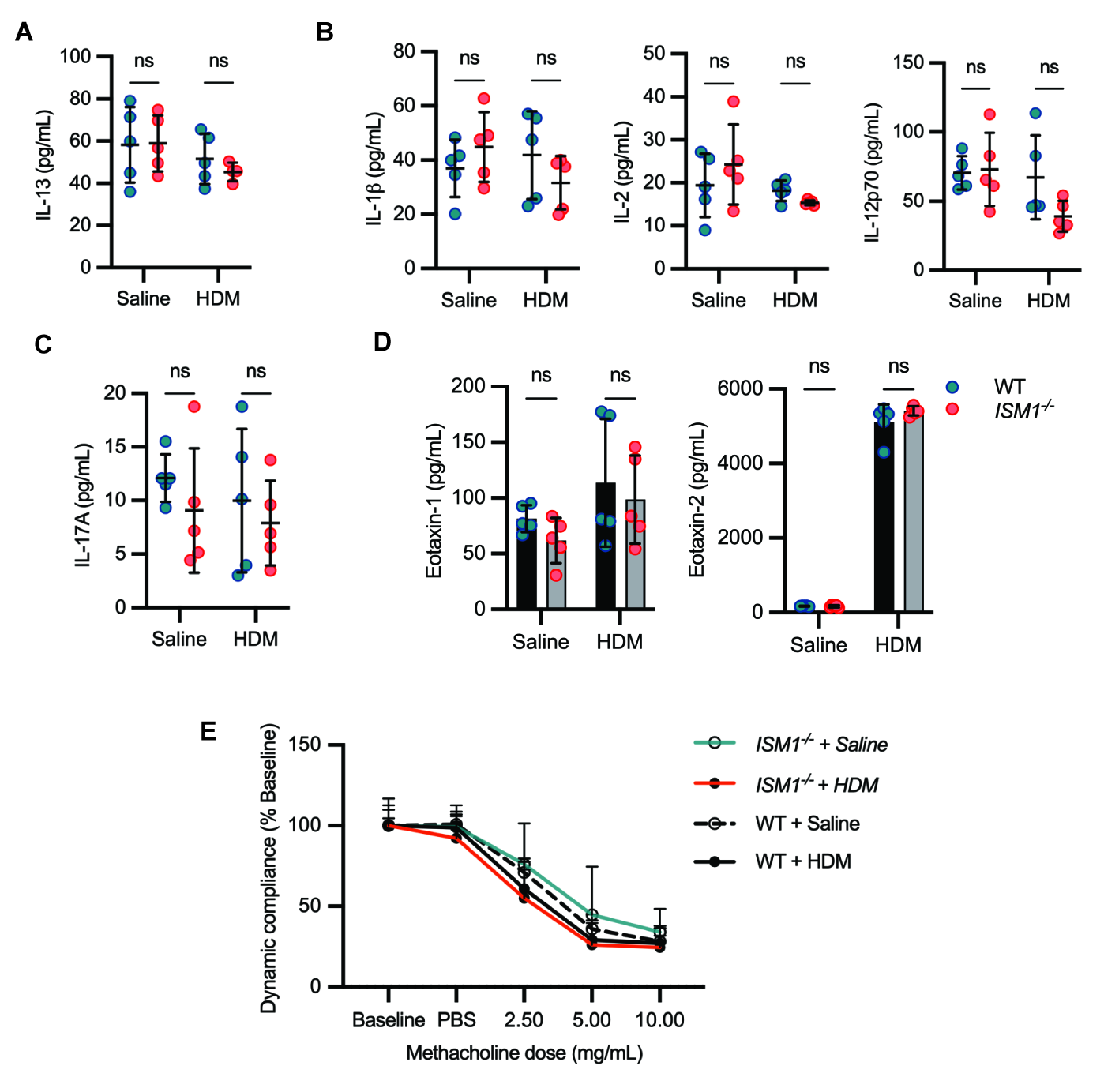

Supplement: Supplementary file 4 — Supplementary Material 4 [file 12931_2023_2569_MOESM4_ESM.tif]

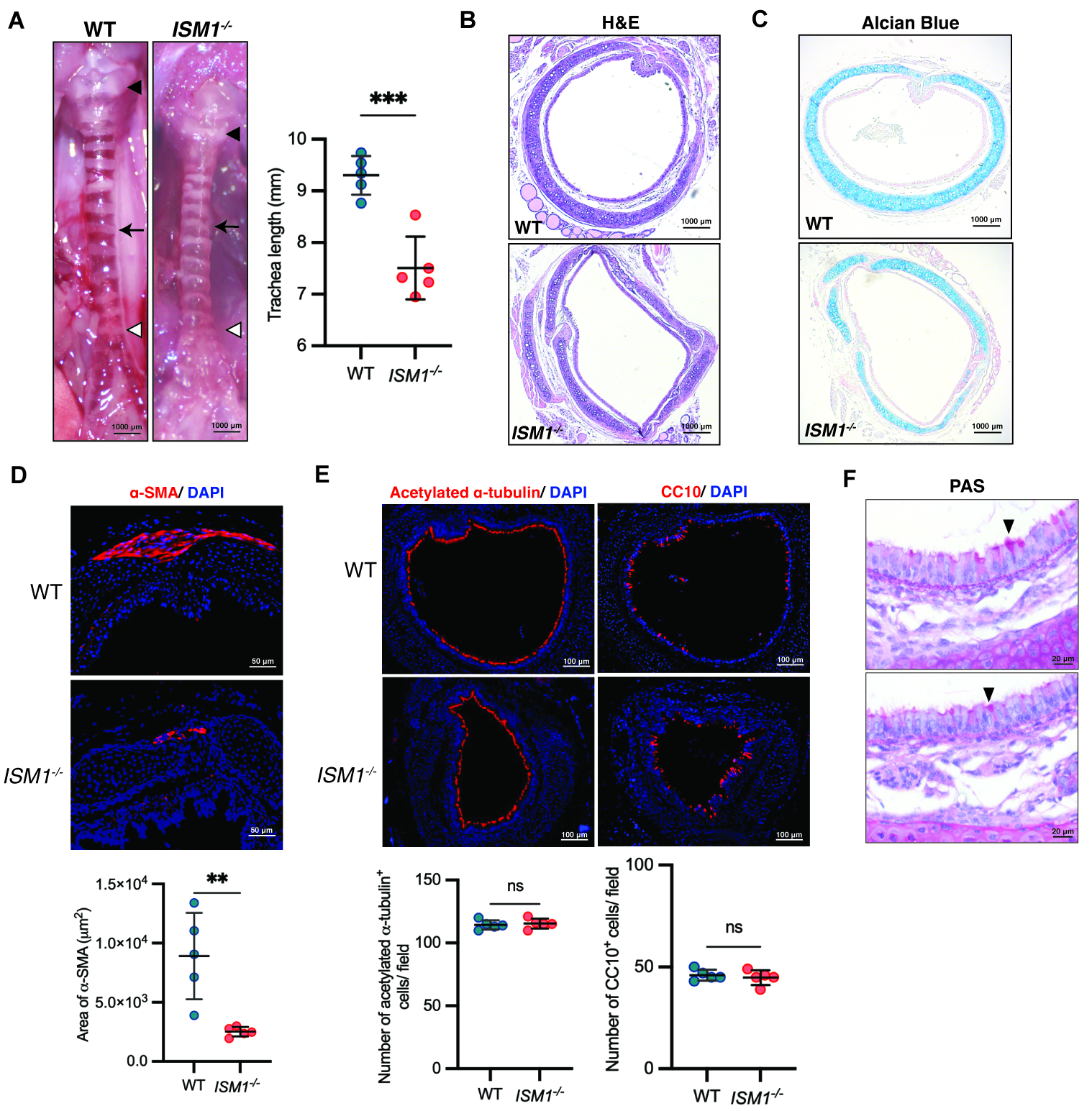

Supplement: Supplementary file 5 — Supplementary Material 5 [file 12931_2023_2569_MOESM5_ESM.tif]

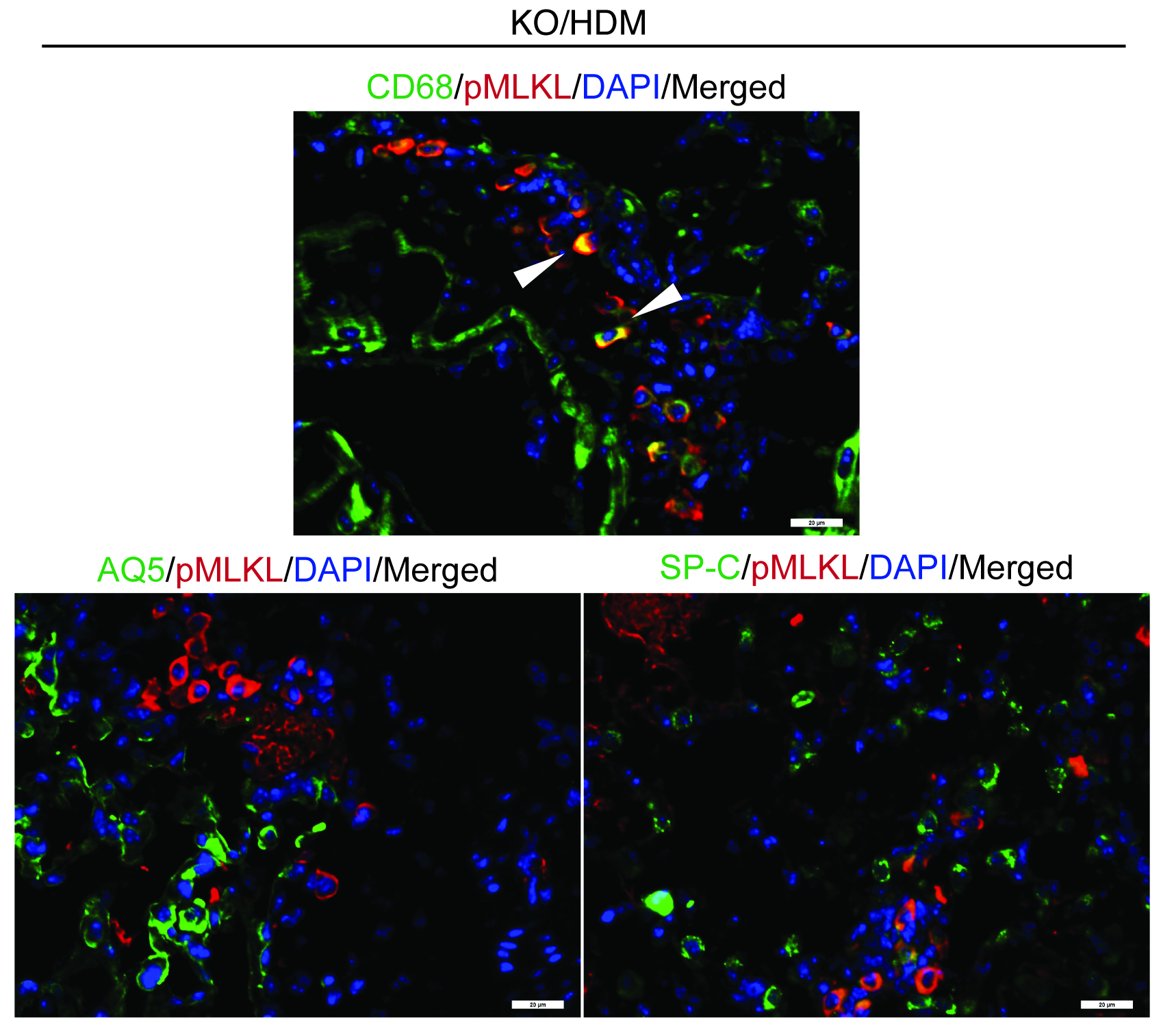

Supplement: Supplementary file 6 — Supplementary Material 6 [file 12931_2023_2569_MOESM6_ESM.tif]

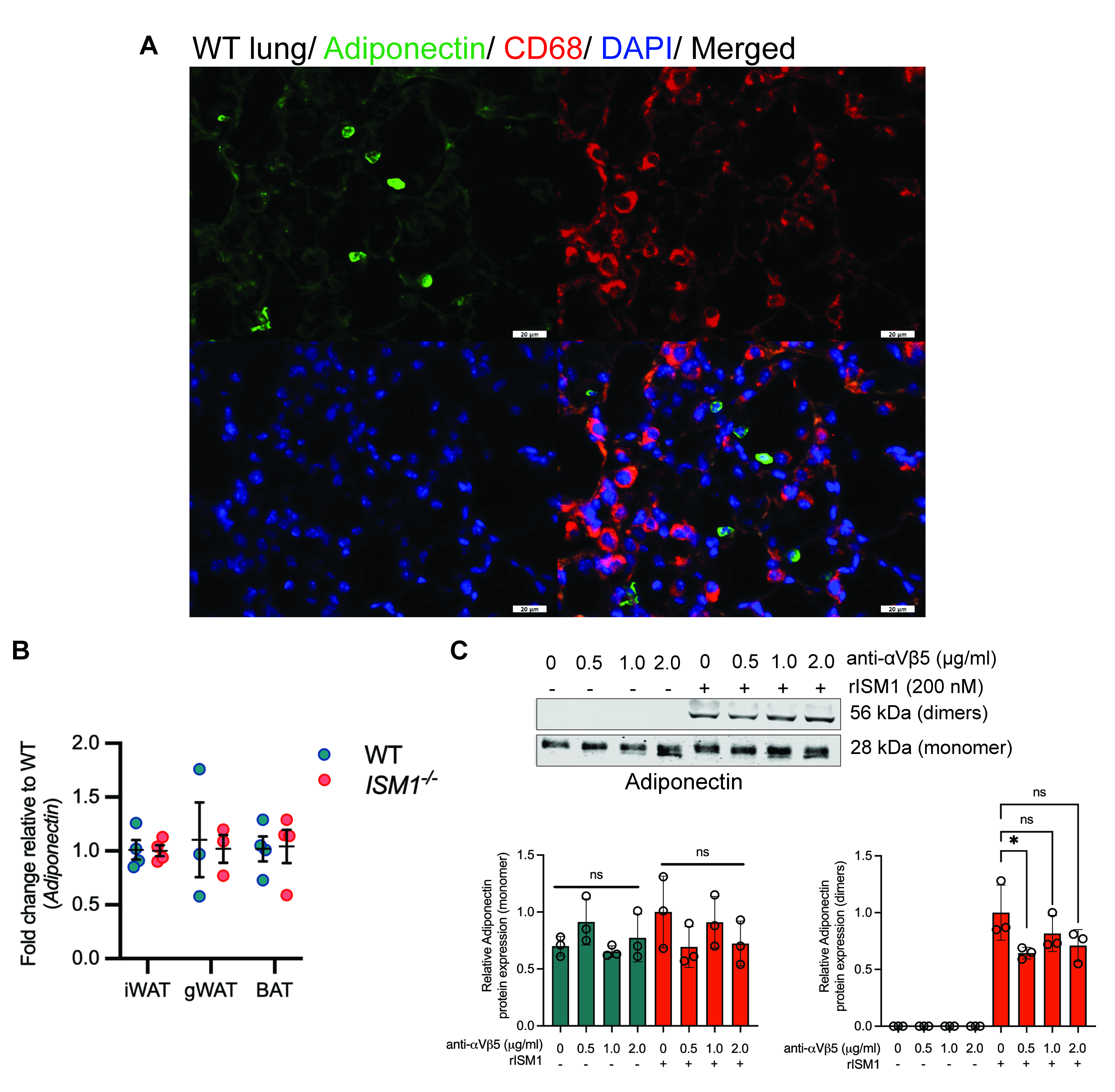

Supplement: Supplementary file 7 — Supplementary Material 7 [file 12931_2023_2569_MOESM7_ESM.tif]

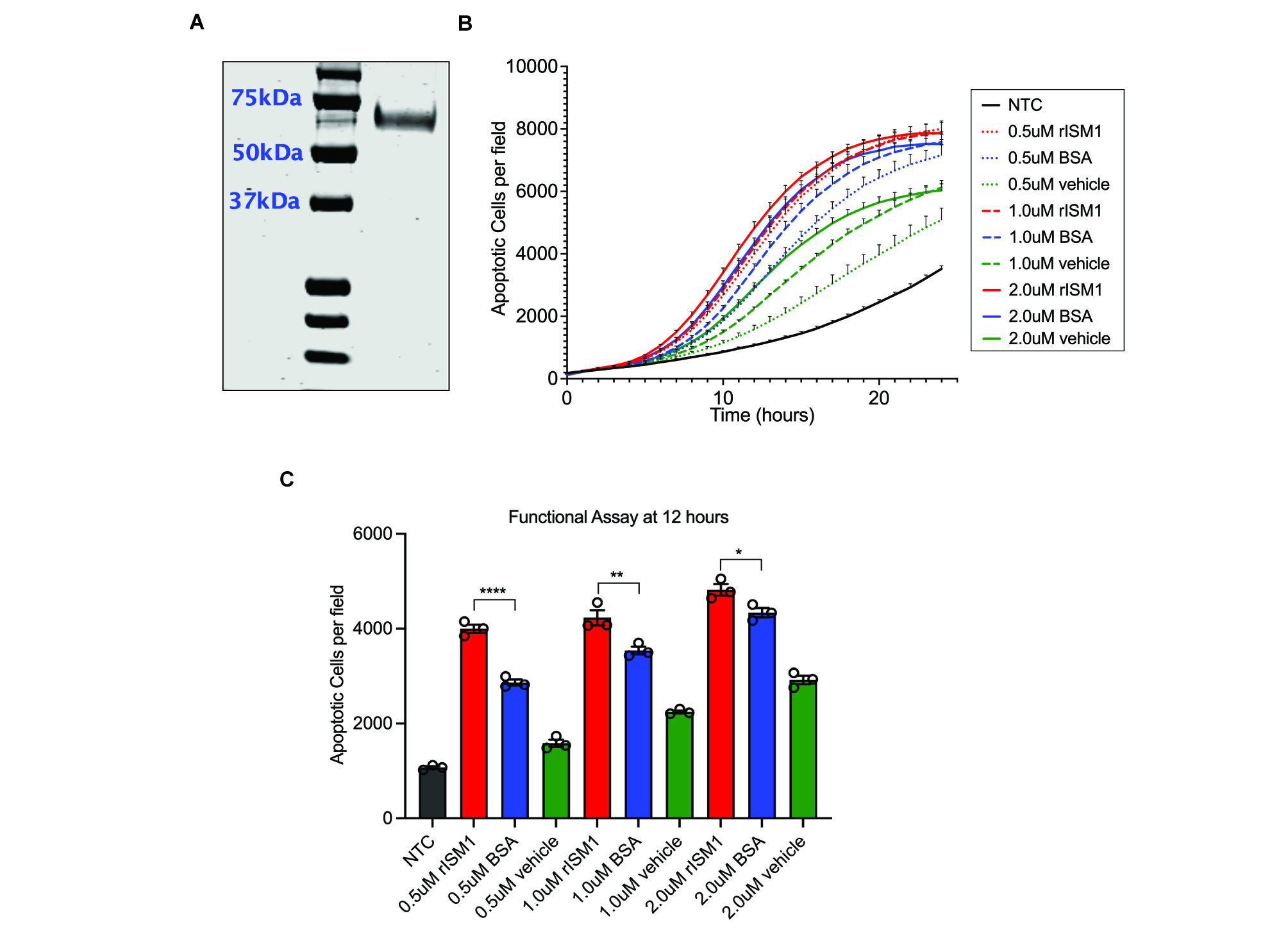

Supplement: Supplementary file 8 — Supplementary Material 8 [file 12931_2023_2569_MOESM8_ESM.tif]

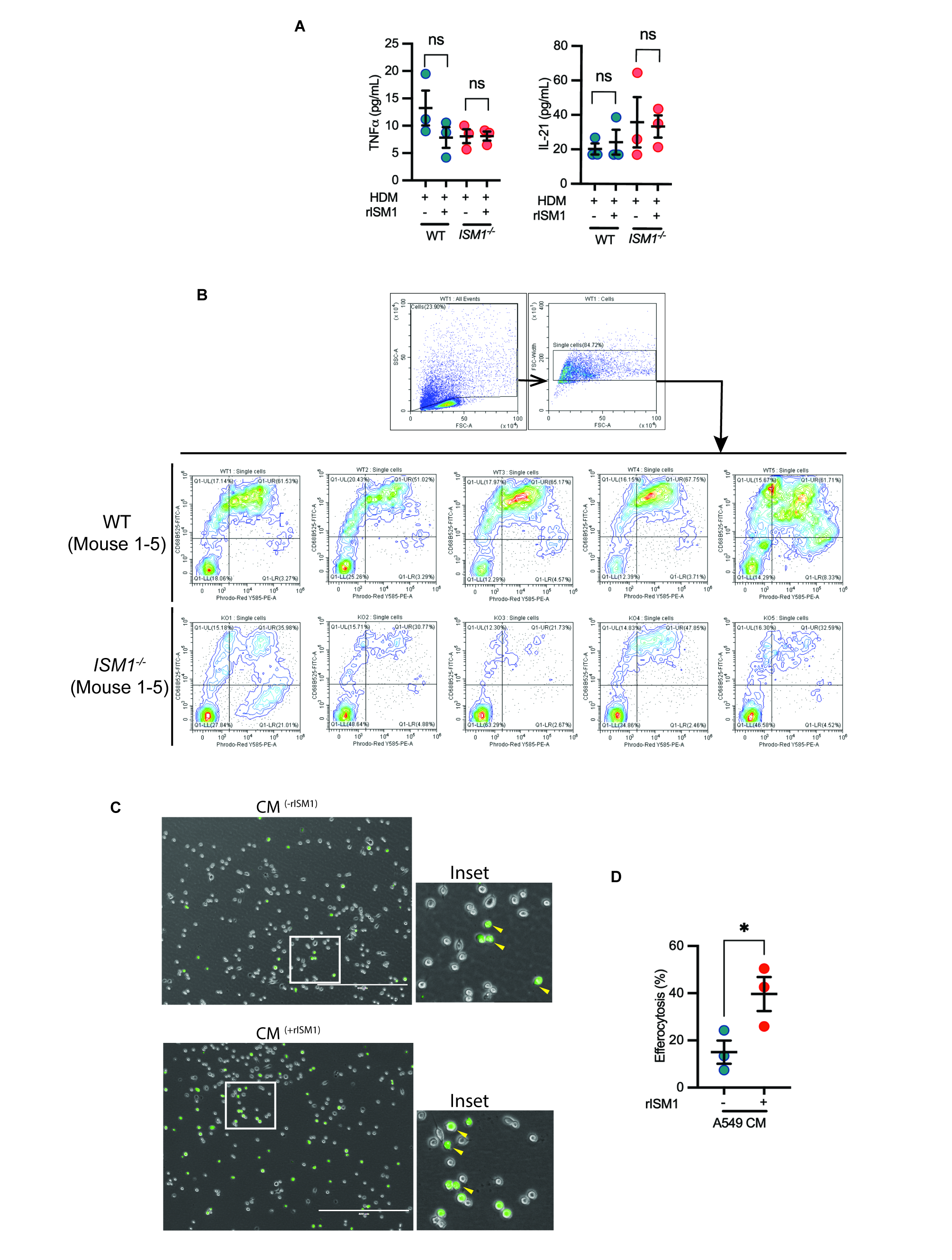

Supplement: Supplementary file 9 — Supplementary Material 9 [file 12931_2023_2569_MOESM9_ESM.tif]
