# Supplementary material for: Isthmin-1 attenuates allergic Asthma by stimulating adiponectin expression and alveolar macrophage efferocytosis in mice
Source: Respir Res. 2023 Nov 6;24:269. doi: 10.1186/s12931-023-02569-1 (PMC10626717; doi:10.1186/s12931-023-02569-1)
Supplement: Supplementary file 10 — Supplementary Material 10 [file 12931_2023_2569_MOESM10_ESM.pdf]

Isthmin-1 attenuates allergic asthma by stimulating adiponectin expression and alveolar macrophage efferocytosis in mice

Jong Huat Tee[1], Udhaya Vijayakumar[1], Mahalakshmi Shanmugasundaram[1], Terence Y. W. Lam[1], Wupeng Liao[2], Yuansheng Yang[3], W. S. Fred Wong[2,4,5]*, Ruowen Ge[1]*

Uncropped blots

Full unedited blot in Figure 2I

Antibodies: Cleaved-TGFβ$_1$ (13kDa) and β-actin (42kDa)

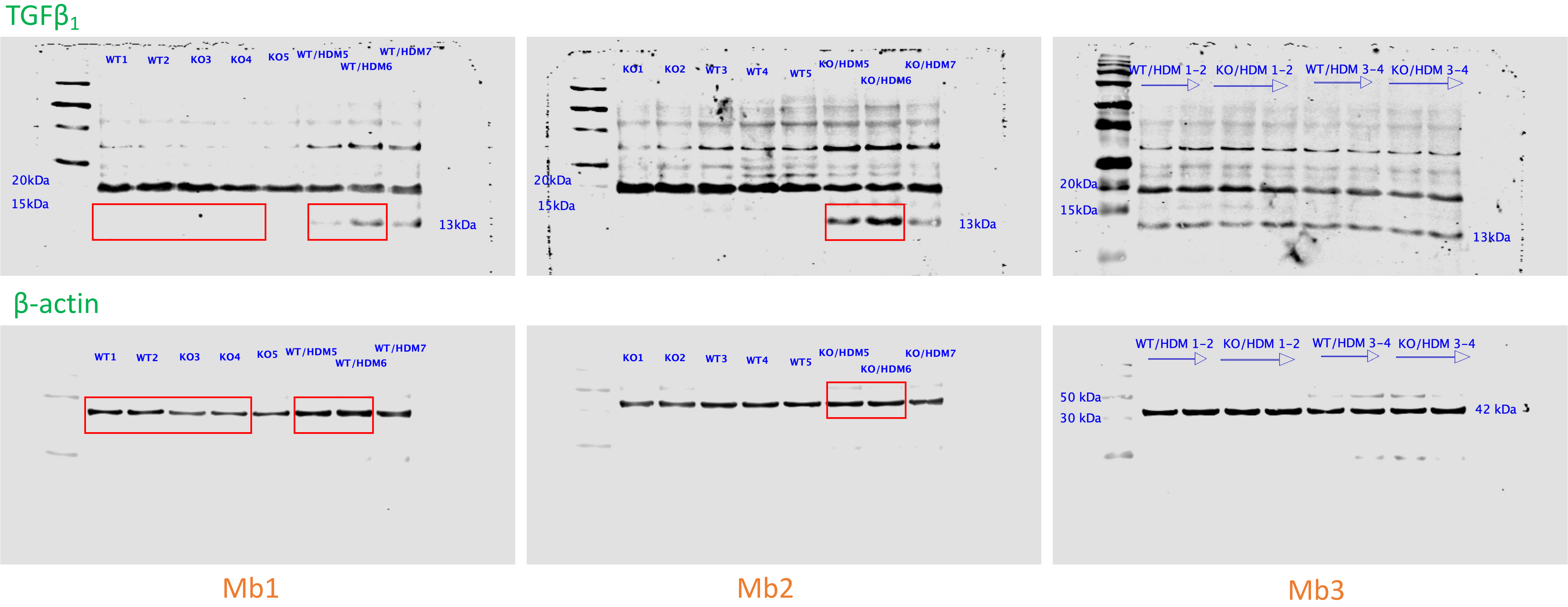

Representative bands

Full unedited blot in Figure 4A

Antibodies: pMLKL (54kDa) and β-actin (42kDa)

Upper band: pMLKL; lower band: β-actin

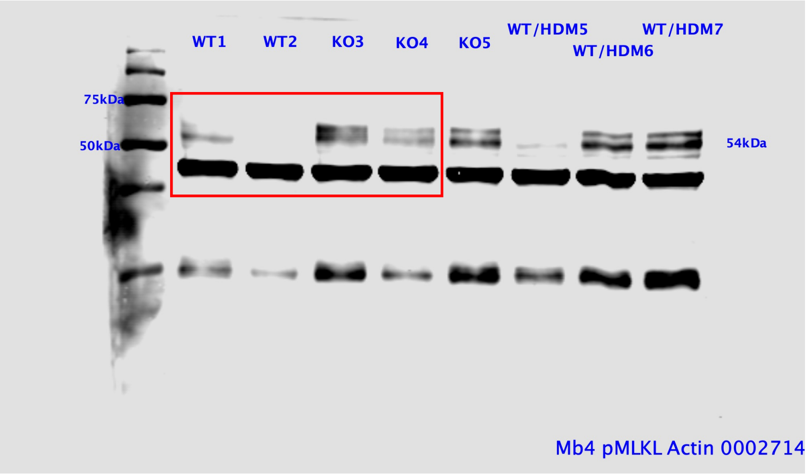

Mb1

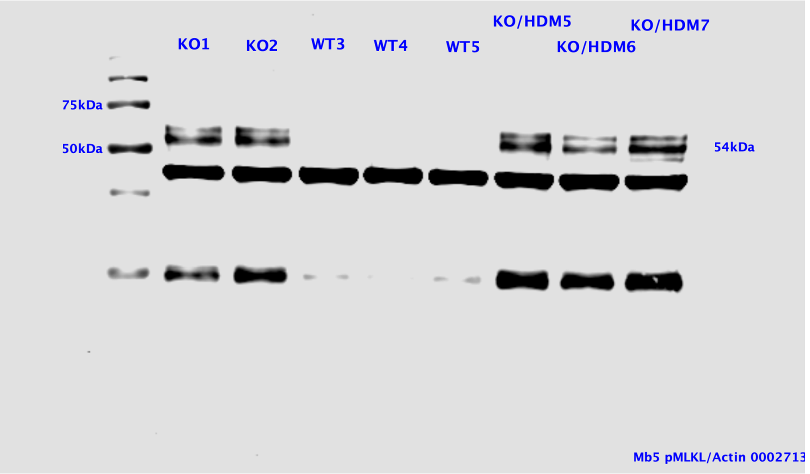

Mb2

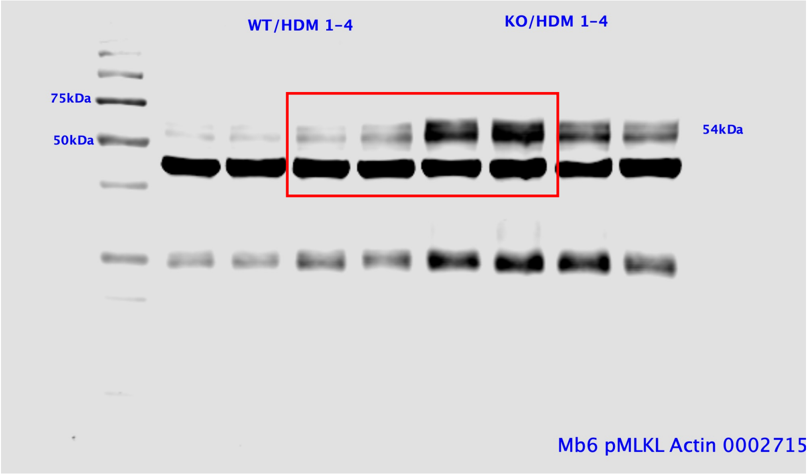

Mb3

Representative bands

Full unedited blot in Figure 4B

Antibodies: RIP3 (57kDa) and β-actin (42kDa)

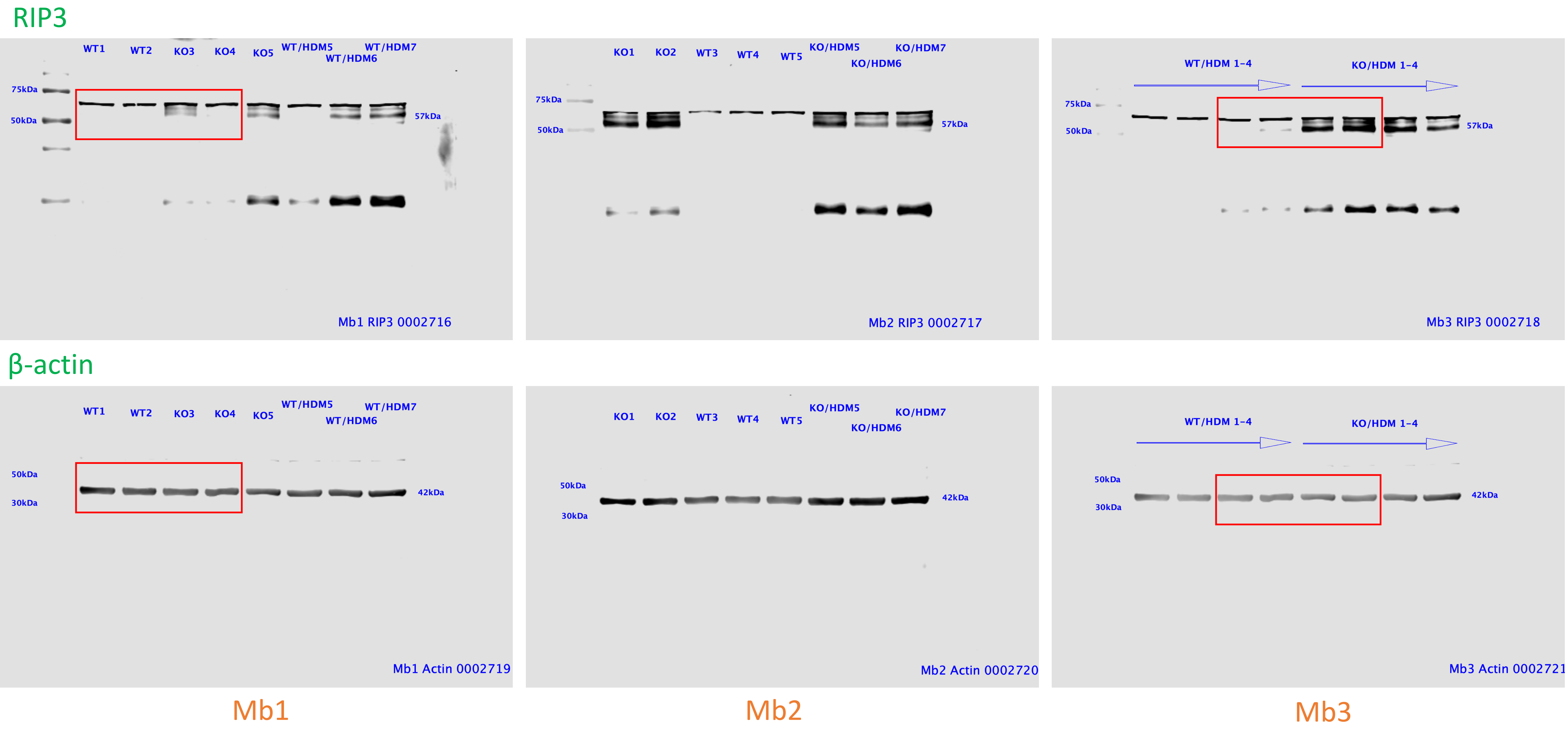

Representative bands

Full unedited blot in Figure 4C

Antibodies: Caspase 8 (57kDa), cleaved Caspase 8 (43kDa) and β-actin (42kDa)

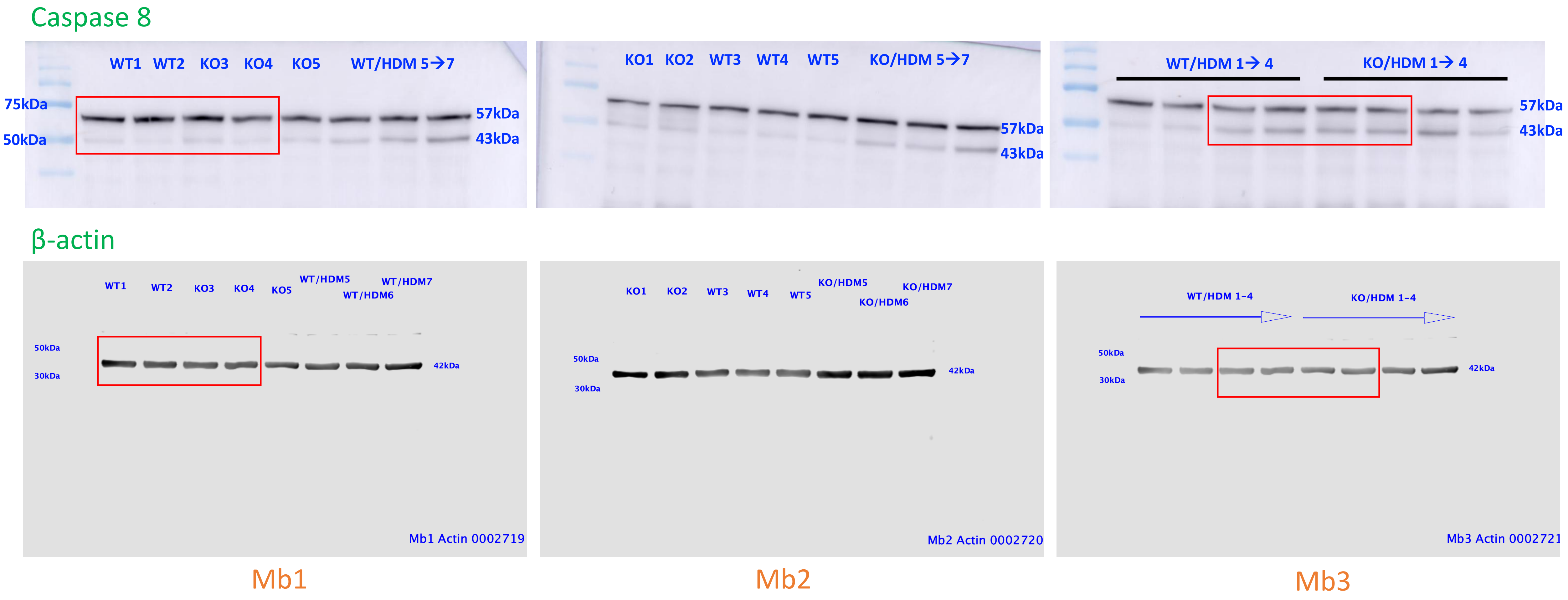

Representative bands

Full unedited blot in Figure 4D

Antibodies: Cleaved Caspase 3 (19kDa) and β-actin (42kDa)

Cleaved Caspase 3

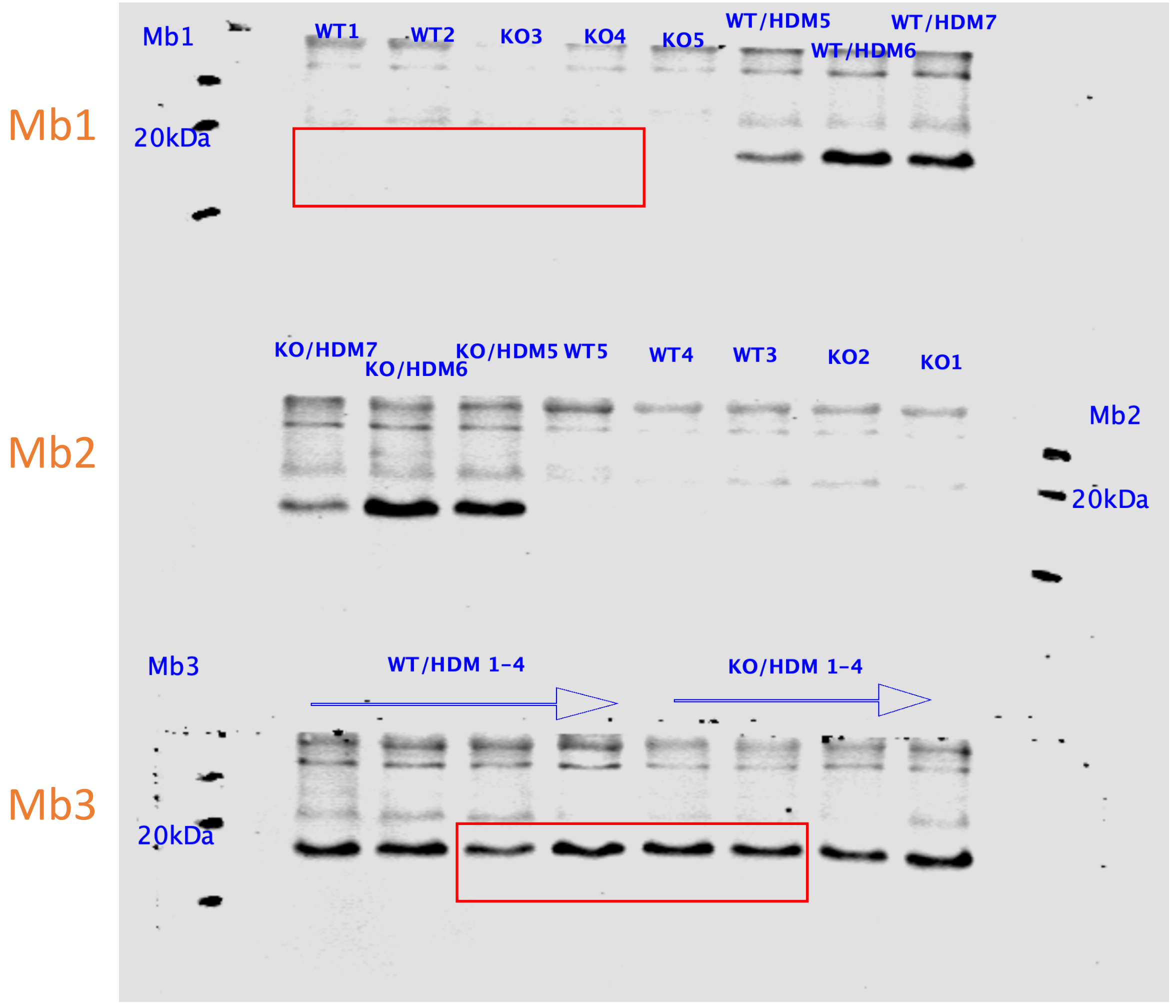

β-actin

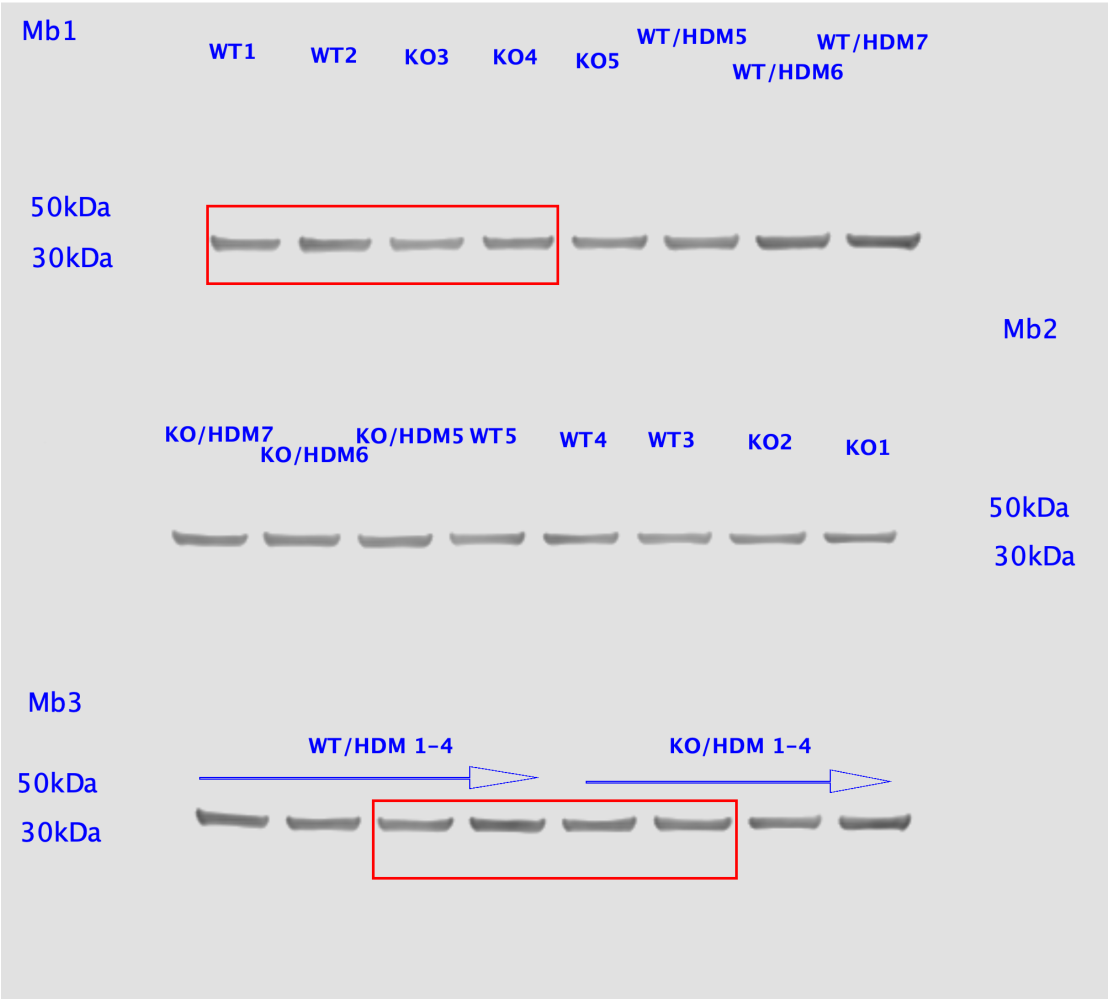

Representative bands

Full unedited blot in Figure 6A

Antibodies: Adiponectin (28kDa) and β-actin (42kDa)

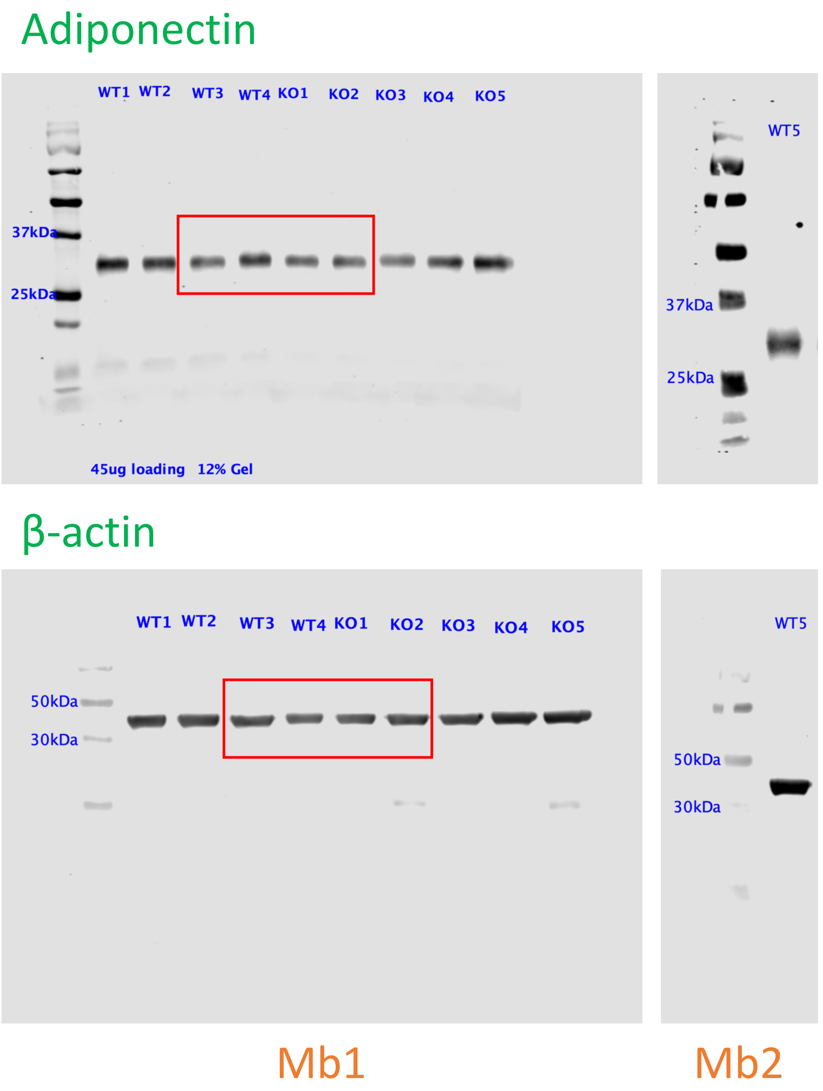

Representative bands

Full unedited blot in Figure 6D
Antibodies: Adiponectin (28kDa)

Adiponectin from conditioned media

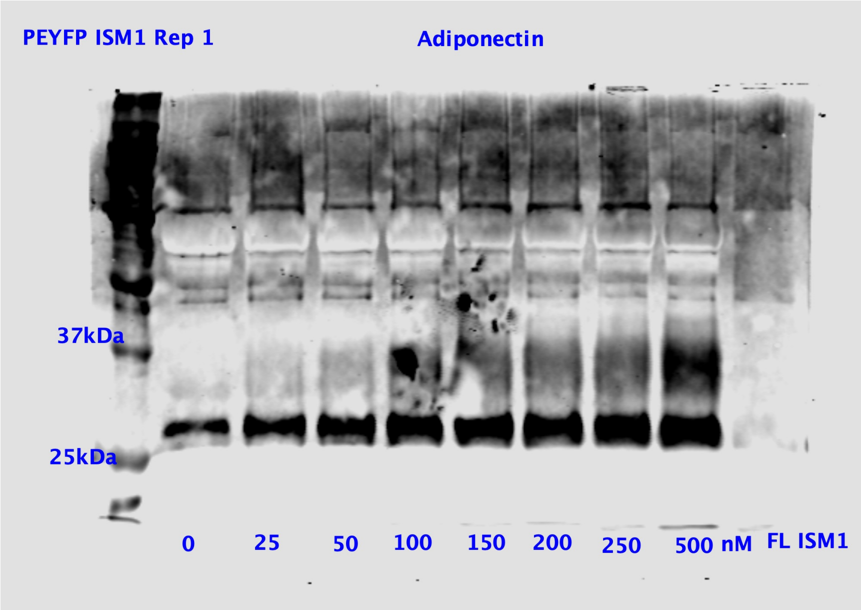

Mb1 (1st repeat)

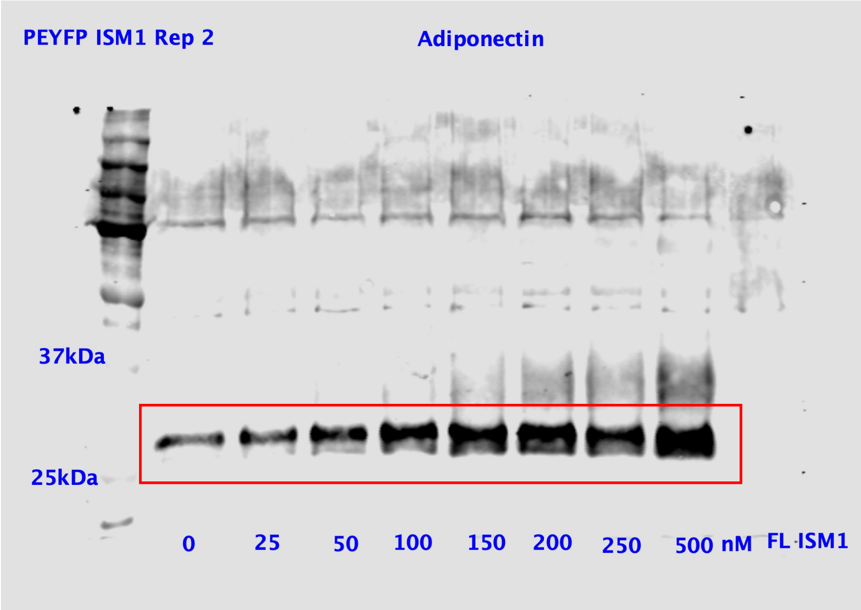

Mb2 (2nd repeat)

Representative bands

Full unedited blot in Figure 6E

Antibodies: PPARγ (66kDa) and β-actin (42kDa)

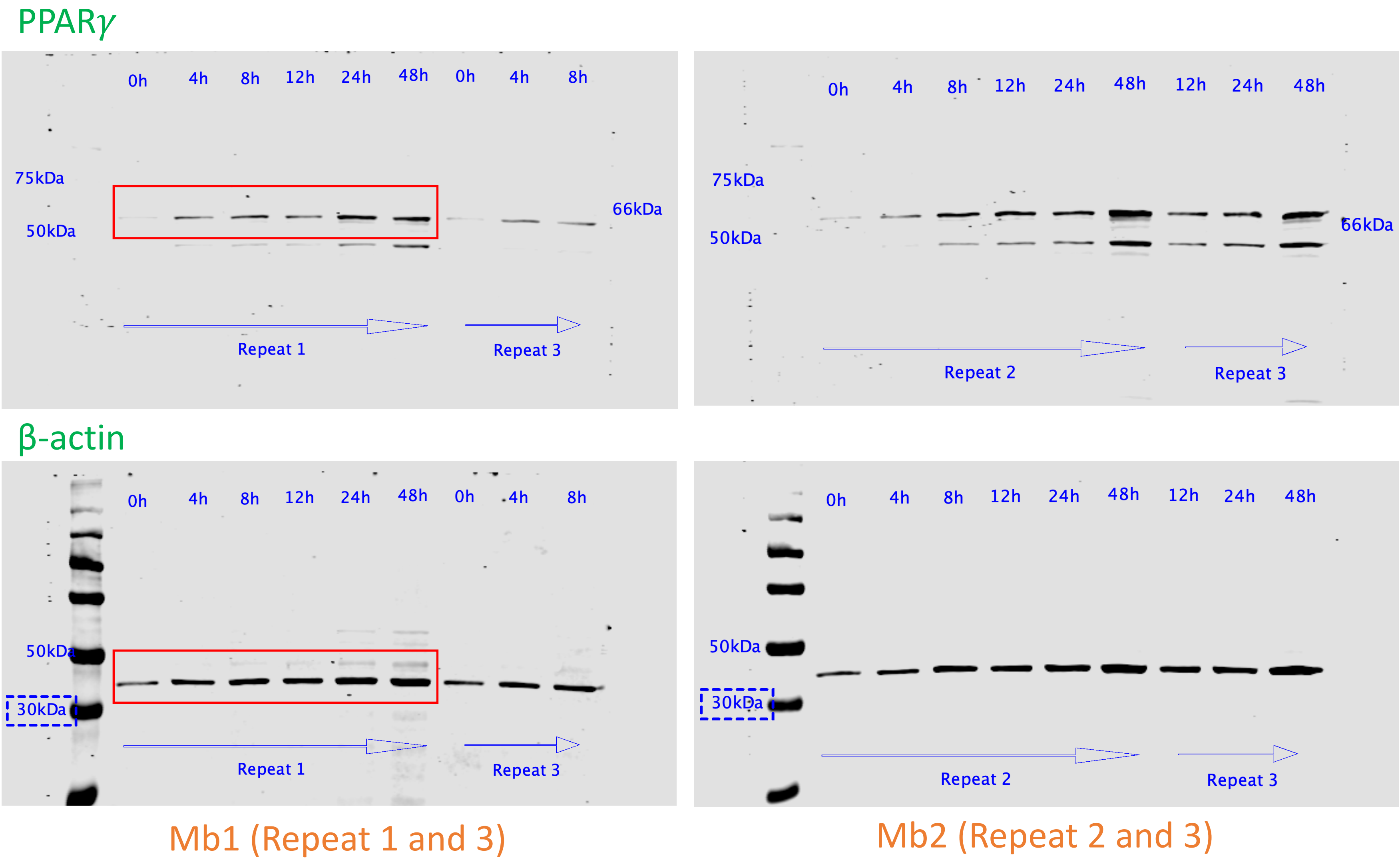

Representative bands

Full unedited blot in Figure 6G

Antibodies: Adiponectin (Dimers:56kDa and Monomers:28kDa)

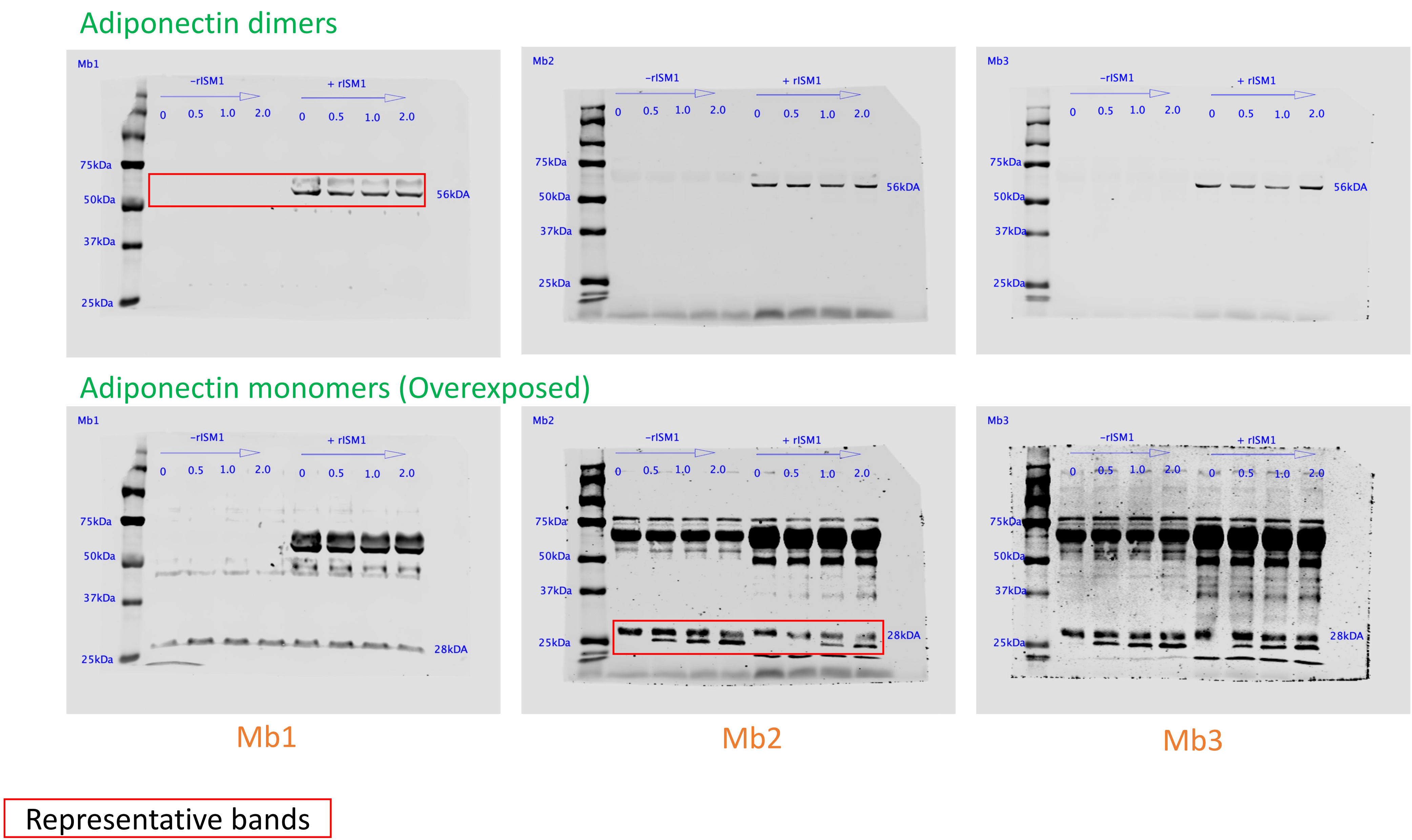

Full unedited blot in Figure 7F

Antibodies: pMLKL (54kDa) and β-actin (42kDa)

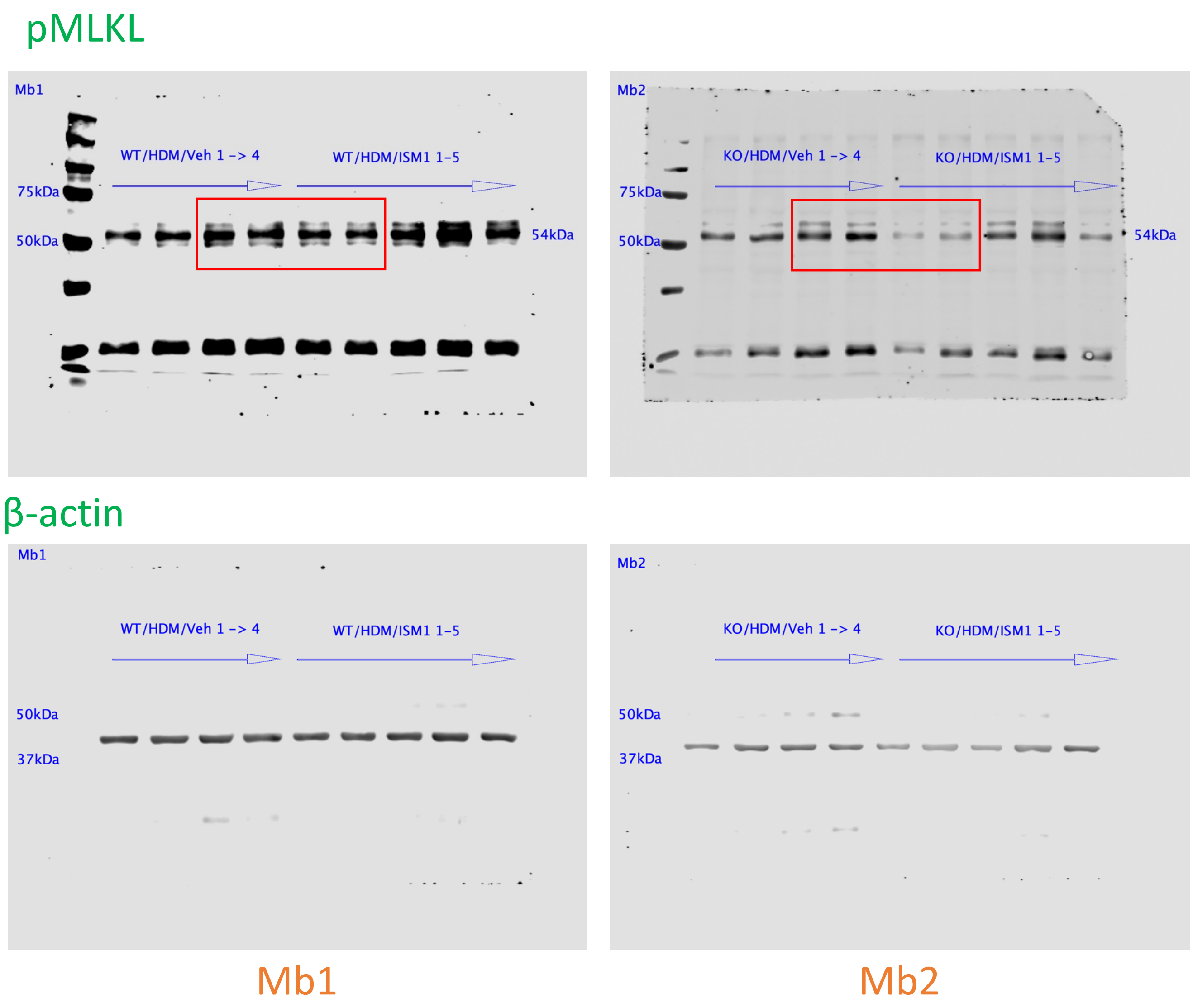

Representative bands
